# The Peptide Ligase Activity of Human Legumain Depends on Fold Stabilization and Balanced Substrate Affinities

**DOI:** 10.1021/acscatal.1c02057

**Published:** 2021-09-10

**Authors:** Elfriede Dall, Vesna Stanojlovic, Fatih Demir, Peter Briza, Sven O. Dahms, Pitter F. Huesgen, Chiara Cabrele, Hans Brandstetter

**Affiliations:** # Department of Biosciences, University of Salzburg, 5020 Salzburg, Austria; § Central Institute for Engineering, Electronics and Analytics, ZEA-3, Forschungszentrum Jülich, 52428 Jülich, Germany; ∥ CECAD, Medical Faculty and University Hospital, University of Cologne, 50931 Cologne, Germany; ⊥ Institute for Biochemistry, Faculty of Mathematics and Natural Sciences, University of Cologne, 50674 Cologne, Germany

**Keywords:** cysteine protease, ligase, transpeptidase, peptide cyclization, chemical surface modification, asparaginyl endopeptidase, pH stabilization

## Abstract

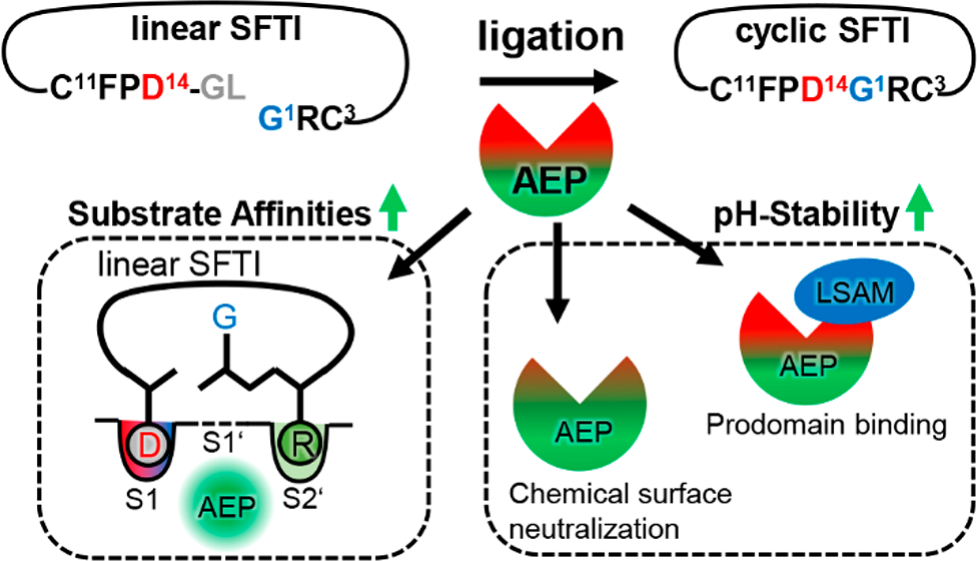

Protein modification by enzymatic breaking and forming of peptide bonds significantly expands the repertoire of genetically encoded protein sequences. The dual protease-ligase legumain exerts the two opposing activities within a single protein scaffold. Primarily localized to the endolysosomal system, legumain represents a key enzyme in the generation of antigenic peptides for subsequent presentation on the MHCII complex. Here we show that human legumain catalyzes the ligation and cyclization of linear peptides at near-neutral pH conditions, where legumain is intrinsically unstable. Conformational stabilization significantly enhanced legumain’s ligase activity, which further benefited from engineering the prime substrate recognition sites for improved affinity. Additionally, we provide evidence that specific legumain activation states allow for differential regulation of its activities. Together these results set the basis for engineering legumain proteases and ligases with applications in biotechnology and drug development.

## Introduction

Mainly localized to the endolysosomal system, the cysteine protease legumain plays important functions for the processing of antigens for presentation on the MHCII complex.^1−3^ Given its specific cleavage after asparagine residues it can develop asparaginyl-endo (AEP) and -carboxypeptidase (ACP) activities in a pH-dependent manner, depending on details of its activation.^4−7^ Human legumain is synthesized as an inactive proenzyme consisting of a caspase-like catalytic domain and a C-terminal death domain-like prodomain (legumain stabilization and activity modulation – LSAM – domain) that are linked by an activation peptide (AP). While single-chain prolegumain and the two-chain ACP state, where the AP was removed, are stable at neutral pH, the isolated active AEP domain is stable only at acidic pH but not at neutral pH.^4^ Furthermore, legumain encodes a peptide ligase activity, that is, legumain can not only hydrolyze peptide bonds but also synthesize them.^8,9^ Protease and ligase activities are in a pH-dependent equilibrium with the protease dominating at acidic pH and the ligase at neutral pH. Although there are numerous studies on the ligase activity of different plant legumain isoforms, only little is known about the ligase activity of mammalian legumain.^10−13^ Until now, ligase activity was demonstrated toward legumain’s physiologic inhibitor cystatin E and the rezymogenization of legumain itself, both resulting in an autoattenuation of its enzymatic activities.^8,9^ In a pathophysiological context, legumain is overexpressed in the majority of human solid tumors, including breast cancer and colorectal cancer.^14,15^ Here, legumain is associated with enhanced tissue invasion and metastasis and consequently overexpression correlates with poor prognosis.^16−18^ Under these pathological conditions, legumain was found translocated to the nucleus, cytoplasm, and extracellular space.^16,18−21^. Recently, there has been growing evidence that legumain is similarly translocated in the aged brain and thereby facilitates aggregation of proteins critically linked with neurodegenerative diseases, finally causing neuronal damage.^19−22^ The presence of active AEP at cellular compartments outside the acidic endolysosome has been puzzling scientists for many years, as it is inconsistent with legumain’s pH-stability profile. Given the fact that legumain ligase activity is predominantly present at near-neutral pH, the relevance of ligase activity for extra-lysosomal legumain is obvious, but it is only scarcely studied until now. Therefore, we set out to structurally and biochemically analyze the ligase activity of human legumain.

## Results and Discussion

### Human Legumain Harbors a pH-Dependent Transpeptidase Activity

**Figure 1:**
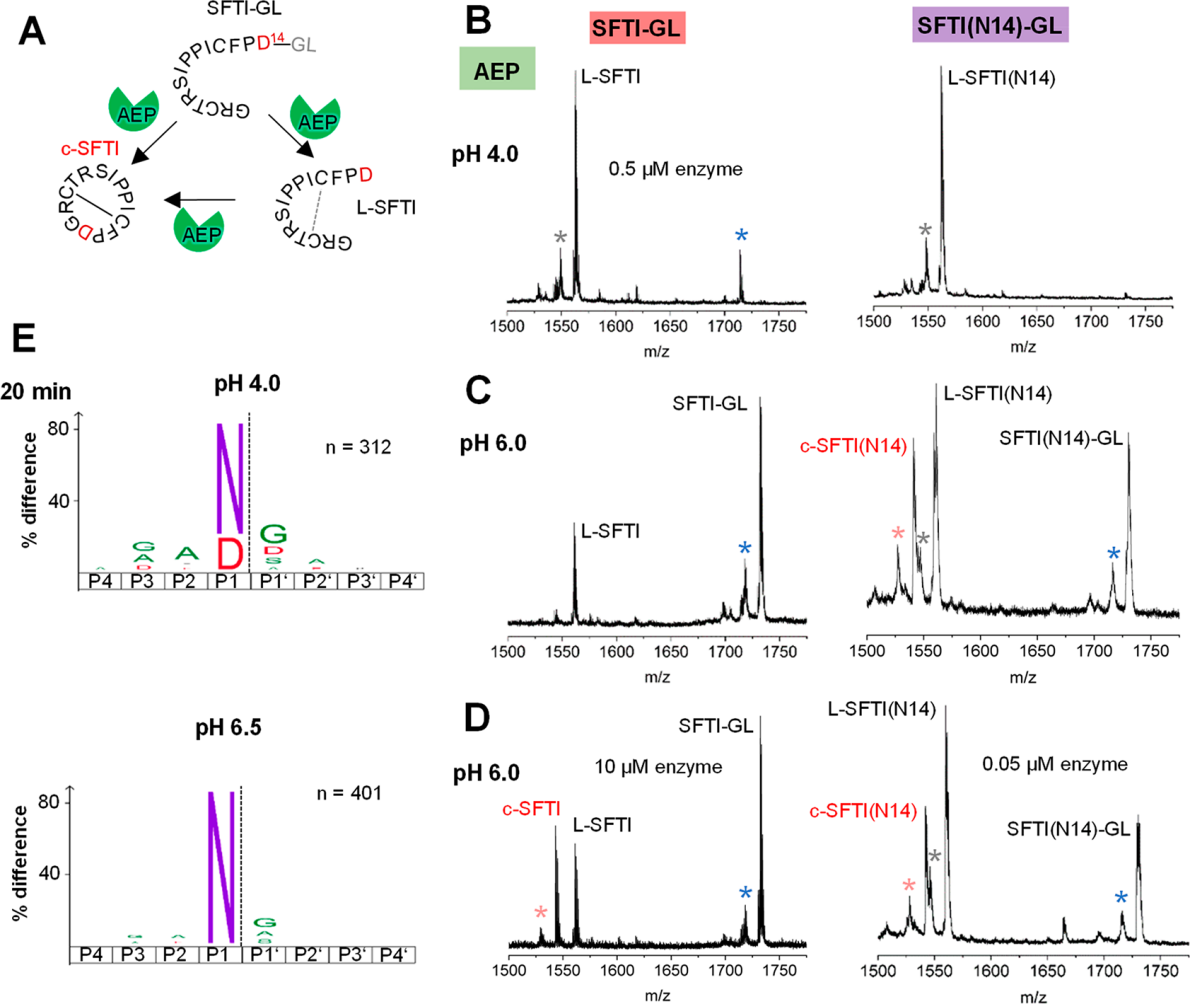
Legumain has protease and transpeptidase activity with specificity for Asn and Asp in P1. (A) Reaction scheme of SFTI-GL precursor (SFTI-GL) cleavage (L-SFTI) and transpeptidation (c-SFTI) catalyzed by legumain. Asp14 serves as the P1 residue after which legumain can cleave and establish peptide bonds. Asp14 is replaced by Asn in the SFTI(N14)-GL variant. SFTI-GL and SFTI(N14)-GL peptides (0.5 mM) were incubated with legumain at 0.5 μM concentration at pH 4.0 (B) or pH 6.0 (C) and the formation of linear L-SFTI peptidase cleavage product and cyclic c-SFTI transpeptidation product was monitored. (D) Same assay as in B but at 10 and 0.05 μM enzyme concentration. A blue star indicates a peak corresponding to demethylated (Δ14 Da) SFTI-GL or SFTI(N14)-GL, respectively. A light-red star indicates a peak corresponding to demethylated (Δ14 Da) c-SFTI or c-SFTI(N14), respectively. This modification may happen at Thr4 or Leu16 and is frequently observed in our MS measurements.^28^ A gray star indicates an unidentified species. (E) IceLogos showing the strict specificity of legumain toward Asn at pH 6.5 and Asn or Asp at pH 4.0 (*p* = 0.05). Small hydrophobic residues are preferred at the positions directly surrounding the P1 residue.

To test if mammalian legumain harbors peptide ligase activity, we established a peptide-cyclization assay based on peptides derived from the sunflower trypsin inhibitor (SFTI) precursor sequence and on mass spectrometry detection. The SFTI is a cyclic inhibitor where cyclization is catalyzed by sunflower legumain mainly via a transpeptidation route.^23−25^ Transpeptidation is supposed to proceed via the formation of a covalent thioester intermediate between the Sγ of the catalytic Cys residue and the carbonyl carbon of the P1-Asp14 residue. Subsequently, the inhibitor is cyclized by the attack of the N-terminus of Gly1, that is, the prime side ligase substrate. Essentially, we used two variants of the precursor peptide, where the P1 residue at position 14 was either Asp (SFTI-GL) as in the original sequence or Asn (SFTI(N14)-GL), which is the preferred legumain protease substrate (Figure 1A). Since thioester formation is an essential prerequisite for transpeptidation to proceed, we extended the precursor peptide by a Gly-Leu sequence, to cover the P1’ and P2’ positions.^26,27^ Indeed, we found that human legumain was able to hydrolyze the precursor peptides at pH 4, leading to the formation of linear L-SFTI and L-SFTI(N14) and confirming the suitability of the peptides as legumain substrates (Figure 1B). However, we did not observe the formation of the cyclic product at acidic pH (Figure 1B and Table S1). Since we knew from previous experiments that ligase activity is favored at near-neutral pH, we repeated the SFTI-cyclization assay at pH 6.0 and 6.5. Indeed, at pH 6, we observed the formation of the cyclic product for both precursor peptides, although with different efficiency (Figure 1C,D; Figures S1, S2, S3; and Tables S1 and S2). While the P1-Asn peptide was hydrolyzed and transpeptidated at 0.5 and 0.05 μM enzyme concentration with a cyclization-to-hydrolysis ratio of 0.7:1 and 0.6:1 and a total of 30% and 25% cyclic product formed, respectively (as based on the relative abundance of the three species: substrate, linear, and cyclic product), the P1-Asp peptide turned out to be a weak protease and transpeptidase substrate at pH 6.0 and higher. Triplicate measurements confirmed the reliability of our measurements (Figure 1, S2 and S3). Although some linear hydrolysis product was present at 0.5 μM enzyme concentration, we did however not observe the formation of cyclic product. Control experiments lacking the enzyme suggest that this linear product may, to some extent, be a result of spontaneous precursor hydrolysis (Figure S2). Only an increased enzyme concentration of 10 μM achieved transpeptidation for the P1-Asp substrate with a cyclization-to-hydrolysis ratio of 1.2:1 and 25% cyclic product formed at pH 6.0, although not at pH 6.5 (Figure S1 and S2). Even though ligation is in principle favored by near-neutral pH conditions, legumain’s stability and its affinity toward P1-Asp substrates decrease with increasing pH value. These effects explain why we observed a reduction in product formation at pH 6.5 when the P1-Asn substrate was used and why we did not observe any hydrolysis and ligation products when the less-favorable P1-Asp substrate was used. This finding was additionally supported by HPLC-MS experiments (Figure S4). As we only observed little hydrolysis of the P1-Asp substrate and since the covalent thioester intermediate is a prerequisite for transpeptidation to work, we concluded that thioester formation must be the rate-limiting step for transpeptidation to occur. To test whether the reaction was really transpeptidation and not ligation, that is, linkage of Gly1 to the free C-terminus of L-SFTI without a thioester intermediate, we further incubated legumain with the control peptides L-SFTI and L-SFTI(N14). As we did not observe conversion to the cyclic product, we concluded that cyclization was mediated by transpeptidation (Figure S1).

### Asparagine Is the Preferred P1 Residue in Peptidase Substrates, over the Whole pH Range

The SFTI-cyclization assay showed that the substrate affinity correlates with transpeptidation efficiency. Therefore, we set out to further investigate legumain’s substrate specificity using PICS experiments.^29^ Specifically, we incubated legumain with a peptide library that was generated by trypsin digestion of an *E. coli* proteome. In line with our SFTI-transpeptidation experiments, at pH 6.5 we found a strong preference for Asn in P1-position and no visible turnover of Asp substrates (Figure 1E). Only at pH 4.0 was Asp accepted to a significant amount and only after a prolonged incubation time (Figure 1E and S5) which is also in excellent agreement with previous reports.^7,30^ Comparison of short and long incubation times further showed that even at pH 4.0, Asn is preferred over Asp (Figure S5). Additionally, small hydrophilic residues were preferentially found in P2, P3, and especially P1’ position, suggesting that substrate affinity can be improved by introducing Gly or Ala in P1’ position. Consistent with this conclusion, glycine is found in both P1’ positions of the SFTI-derived precursor peptide.

### Crystal Structure of Legumain in Complex with Ligase Substrate Uncovered Equilibrium between Protease and Ligase States of the Catalytic Cys189

**Figure 2:**
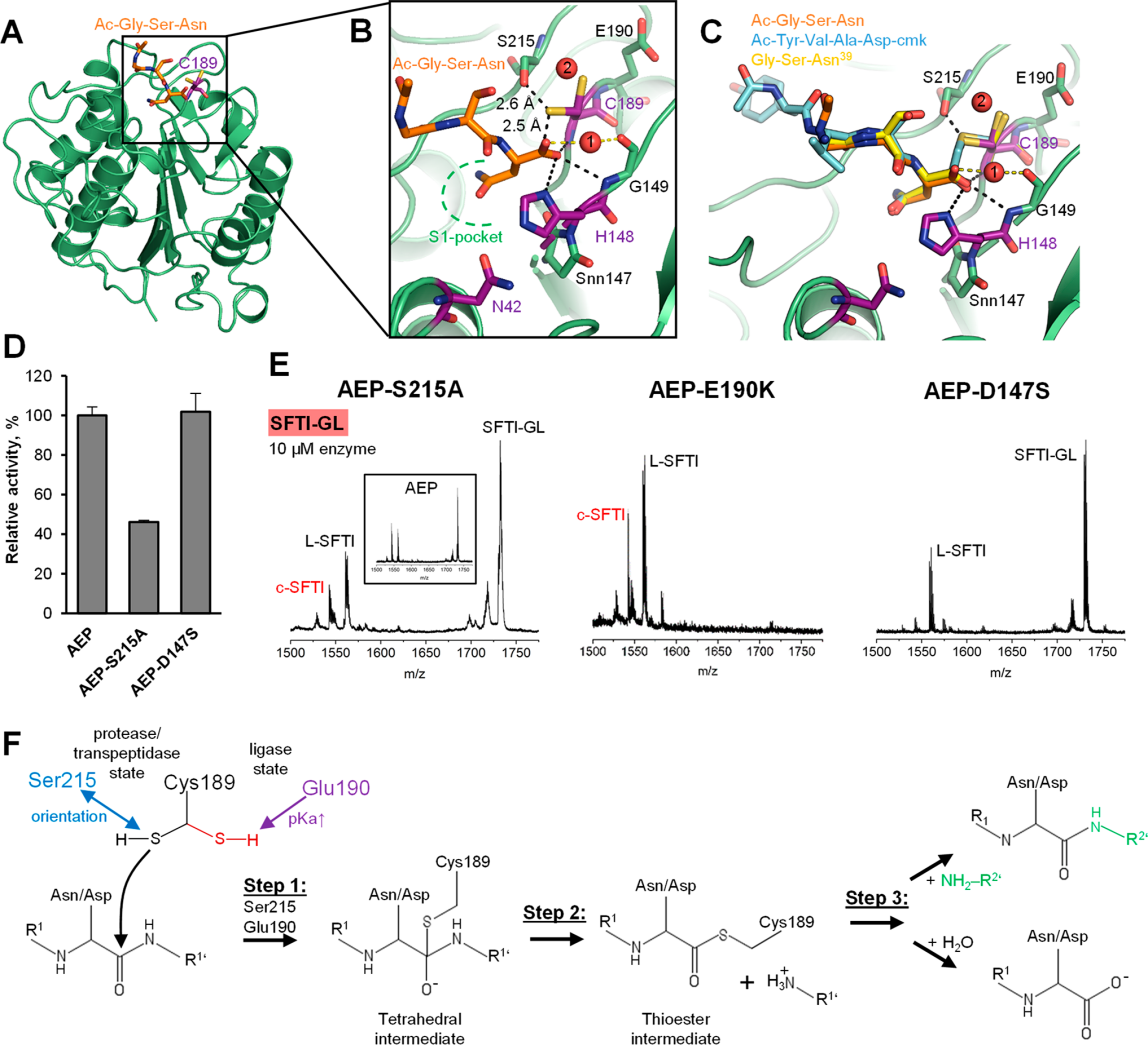
Crystal structure of legumain in complex with ligase substrate. (A) Cartoon representation of legumain (green) bound to the Ac-Gly-Ser-Asn (orange carbons) tripeptide. The catalytic Cys189 is shown in purple sticks. (B) Zoom-in view on the active site of legumain bound to the Ac-GSN peptide. Catalytic residues are shown in purple, residues with regulatory function in green sticks and water molecules as red spheres. (C) Superposition of legumain bound to Ac-Gly-Ser-Asn, Ac-Tyr-Val-Ala-Asp-cmk (pdb 4aw9), and the reactive center loop Gly-Ser-Asn^39^ of human cystatin E (pdb 4n6n). (D) Reactivity of wild-type legumain, and the S215A and D147S variants toward the AAN-AMC substrate, assayed at pH 5.5. (E) Transpeptidation of the SFTI-GL peptide by the S215A, E190K, and D147S variants of legumain, assayed at pH 6.0 and 10 μM enzyme concentration. (F) Protonation of Cys189 is regulated by Glu190, which is stabilizing the ligase state of Cys189. Ser215 helps in preorienting and stabilizing the Cys189 side-chain in the protease/transpeptidase state of legumain. In step 1 of a hydrolysis or transpeptidation reaction, the Cys189 Sγ is attacking the scissile peptide bond, leading to the formation of a tetrahedral intermediate. In step 2, the primed product is released, leading to the thioester intermediate. In Step 3 of the reaction, the intermediate is either released by the catalytic water molecule (hydrolysis) or by the N-terminus of a close by prime side substrate R^2^’ (transpeptidation). Snn147 favors the transpeptidation pathway.

To better understand how ligase and protease/transpeptidase activities are simultaneously implemented in the legumain active site, we solved the crystal structure of human legumain in complex with a model substrate (Table S3). Specifically, we synthesized the tripeptide Ac-Gly-Ser-Asn^39^ (GSN), based on the reactive center loop (RCL) sequence of human cystatin E, which is binding to the legumain active site and is known to be cleaved and ligated between the Asn^39^-Ser^40^ (P1–P1’) scissile peptide bond. The Ac-Gly-Ser-Asn peptide therefore mimics the non-prime protease cleavage product as well as the ligase substrate. Overall, the structure of legumain in complex with the GSN peptide looked similar to other structures we previously solved (Figure 2A). No major structural changes had occurred upon binding of the tripeptide. Similar to other structures of human legumain, we observed a cluster of negatively charged amino acids surrounding the substrate binding sites, referred to as the electrostatic stability switch, ESS.^4^ The legumain bound conformation of the tripeptide was very similar to the one we observed for the covalent Tyr-Val-Ala-Asp-cmk (YVAD-cmk) inhibitor and the RCL on human cystatin E (Figures 2B,C and S6).^31^ We found the P1-Asn residue bound to the S1 pocket and its carboxy-terminus stabilized in the oxyanion hole formed by amide nitrogens of Gly149, Cys189, and the imidazole side chain of His148. Additionally, we found a water molecule in close proximity to the oxyanion hole, which was coordinated by the carboxyl-groups of Gly149 and the P1-Asn residue. Because of its localization close to the substrate, it will likely represent the catalytic water, which is essential for hydrolysis of the thioester intermediate during protease activity. Furthermore, we found that the side chain of the catalytic Cys189 adopted two distinct conformations with equal occupancy. While one orientation was pointing toward the scissile peptide bond, the second conformational variant was rotated by approximately 90°. On the basis of previous observations,^8^ we concluded that the orientation pointing toward the scissile peptide bond shows the protease and transpeptidase state, while the orientation pointing away from the scissile peptide bond represents the ligase state of human legumain. This was the first time that we could show both states coexisting in the same crystal structure. These alternate states suggest an intrinsic equilibrium between protease/transpeptidase and ligase states of legumain. Since the ligase activity of legumain is dominant at near-neutral pH, the ligase state might be favored at higher pH. Indeed, while the present structure was solved at acidic pH (pH 4.5), where we see an equal distribution between both states, the structure of legumain in complex with cystatin E was solved at pH 6.5,^8^ where we see the ligase orientation dominating. Furthermore, we found the side chain of Ser215, which is in close proximity to the catalytic Cys189, oriented toward the Cys Sγ in the protease state (Figure 2B,C). Both side chains are in hydrogen-bonding distance, suggesting that Ser215 is a previously unrecognized regulator of protease/transpeptidase activity. The Ser215 is reminiscent to Ser214 in chymotrypsin-like proteases, which completes the catalytic residues to form a catalytic tetrad.^32^ Besides Ser215, we also identified Glu190 and Asp147 as potential regulators of legumain ligase and transpeptidase activities because of their localization close to the catalytic Cys189 and His148 residues (Figure 2B,C and S7). Asp147 forms a conserved succinimide (Snn147) in legumain.

### Ser215 and Snn147 Are Critical Regulators of Legumain Transpeptidase Activity

To test the relevance of Ser215, Glu190, and Snn147 for legumain activities, we prepared S215A, Glu190K, and D147S point mutants. Using the Z-Ala-Ala-Asn-AMC (AAN-AMC) substrate, we found that the S215A mutant showed an approximately 50% reduction in protease activity, confirming the relevance of this residue for protease activity (Figure 2D). When testing the transpeptidase activity of this mutant, we observed a slight reduction in precursor turnover of both precursor peptides as compared with the wild-type enzyme, a reduction of the cyclization-to-hydrolysis ratio to 0.6:1 (SFTI-GL) and 0.3:1 (SFTI(N14)-GL), and only approximately 10% of cyclic product formed (Figure 2E and S6C). These effects confirm the relevance of this residue for transpeptidase activity. The E190K mutation led to a significant increase in legumain protease activity^4^ and SFTI-GL hydrolysis and cyclization with approximately 40% (P1-Asp) and 30% (P1-Asn) product formed (Figure 2E), even though the cyclization-to-hydrolysis ratio was reduced (0.7:1) or similar (0.6:1) for the P1-Asp and P1-Asn precursor peptides, respectively. Similar to the p*K*_a_ tuning effect of the E190K variant,^4^ we propose that Ser215 will boost legumain protease and transpeptidase activities by stabilizing and preorienting the catalytic Cys189 thiol in a productive conformation. Possibly, Ser215 may also favor the deprotonation of Cys189 Sγ and thereby facilitate the formation of the thioester intermediate.^33^ In the opposite way, Glu190 favors the protonated form of Cys189 and thereby negatively affects the formation of the thioester intermediate. Taken together, we suggest that Ser215 and Glu190 implement a balanced on–off switch that regulates the protonation and orientation of Cys189 and therefore the activity of legumain (Figure 2F). Indeed, Ser215 is conserved in the majority of known legumain sequences (Figure S7).

Previously, we could show that the Snn147 residue is critical for ligation of human cystatin E.^8^ This was confirmed by a D147S mutant, which was not able to ligate human cystatin E. Using the AAN-AMC substrate, we could further show that this mutant has similar proteolytic activity as wild-type legumain (Figure 2D), which, in turn, confirms the correct architecture of the non-prime substrate binding sites and the active site. When we tested the D147S mutant toward the SFTI precursor peptides, we observed precursor hydrolysis for both P1-Asn and Asp variants (Figure 2E and S6C), showing that the peptide could properly bind to the active site of the D147S mutant. However, in contrast to wild-type legumain, we did not observe formation of the cyclic product for the P1-Asp substrate and only about 10% product formation for the P1-Asn substrate, further demonstrating the relevance of this residue for the transpeptidation reaction. Since the D147S variant showed wild-type-like protease activity, we suggest that Snn147 is not directly interfering in step 1, the thioester formation. However, as the transpeptidase activity of the D147S mutant was significantly reduced (or abolished), we suggest that Snn147 primes legumain toward transpeptidase (and ligase) activity by favoring the aminolysis of the thioester intermediate over hydrolysis (step 2, Figure 2F). In contrast, the D147S mutant may favor product release by the catalytic water rather than by the prime side N-terminus. In agreement with this observation and because of its location, Snn147 may have a regulatory effect on the substrate or indirectly, on the prime substrate binding sites.

To further analyze the phylogenetic relevance of active site residues for legumain activity, we used CoeViz to do a covariance analysis (Figure S8). On the basis of χ^2^ scores, the catalytic residues Cys189 and His148 and additionally Gly149, which forms the oxyanion pocket, are clustered together (Figure S8A). Interestingly Asn42, which we previously identified as the third residue of the catalytic triad in legumain-like proteases, did not appear in the same cluster as Cys189 and His148, confirming that Asn42 is specific to legumain-like proteases and not present in the caspases. Similarly, when we reviewed the closest relationships for residue His148 after applying a ≥ 0.3 cutoff to χ^2^ scores, Cys189 and Gly149 appeared on the diagram (Figure S8B). Interestingly, when we analyzed the closest relationships of Ser215, we identified Asp147 and Glu190 as closest relatives (Figure S8C). Covariance analysis therefore further suggests that together Ser215, Asp147, and Glu190 form a cluster of regulatory residues in legumain-like proteases. This is in nice agreement with our experimental data showing that these three amino acids are critical regulators of legumain’s protease and ligase activities.

### The S2’ Pocket Is Critical for Transpeptidase Activity

**Figure 3:**
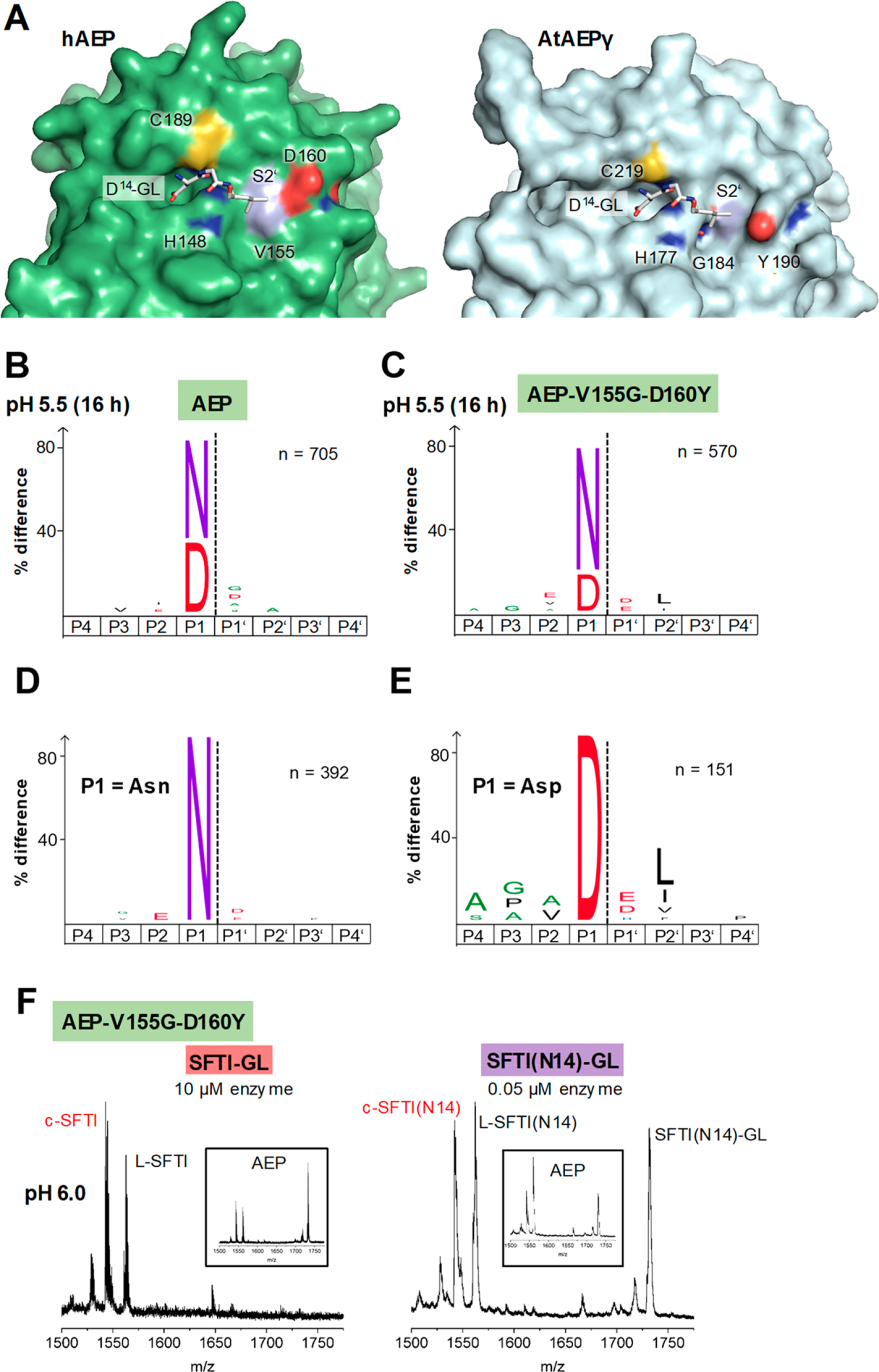
The S2’ pocket is critical for transpeptidase activity and differs in plant and mammalian legumain. (A) Surface representation of human legumain (pdb 4aw9) and AtAEPγ (5obt) showing the active site in complex with an Asp-Gly-Leu peptide (white sticks). Binding of the peptide was modeled based on the structure of legumain in complex with cystatin E (4n6o). (B) IceLogos of human wild-type legumain (AEPwt) and the AEP-V155G-D160Y mutant (C) after 16 h incubation at pH 5.5 show a preference for Leu in P2’ for the double mutant (*p* = 0.05). (D) IceLogo of AEP-V155G-D160Y after selecting peptides harboring Asn in P1 position. (E) IceLogo of AEP-V155G-D160Y after selecting peptides harboring Asp in P1 position. The preference for Leu in P2’ position is especially prominent for peptides harboring Asp in P1 position. (F) SFTI-GL and SFTI(N14)-GL cyclization assay using the AEP-V155G-D160Y mutant.

To understand differences in transpeptidase activity between different legumain-like proteases, we prepared models where we docked the Asp^14^-Gly-Leu (P1-P1’-P2’) tripeptide derived from the SFTI precursor sequence to the active sites of human legumain and *A.th*. legumain isoform γ (AtLEGγ; Figure 3A and S9A). Interestingly, we observed significant differences especially on the prime substrate binding sites. While AtLEGγ has a deep and pronounced S2’ pocket, human legumain has a flat prime side. The bottom of the S2’ pocket is formed by Gly184 in plant legumains, which is replaced by a bulkier Val155 in human legumain (Figure 3A and S7,9). The eastern wall is formed by Tyr190 (AtLEGγ numbering) in plants, corresponding to Asp160 in mammals. We hypothesized that these differences in the prime side architecture would result in different affinities to the SFTI precursor peptide. Our models suggested that the Asp^14^-Gly-Leu sequence would ideally fit to the S2’ site of AtLEGγ, while it should exhibit limited interactions with human legumain. Indeed, this hypothesis nicely fits with our previous data showing about 70% cyclic SFTI product formed with AtLEGγ^26^ and only about 25% with human legumain.

### Increasing Prime-Side Substrate Affinity Enhances Transpeptidase Activity

Following the observation that the S2’ pocket is critical for transpeptidase activity, we aimed to generate a better transpeptidase by grafting the S2’ pocket of plant legumains on human legumain. To that end, we prepared V155G and V155G-D160Y legumain mutants. Using PICS experiments, we found that the V155G-D160Y variant indeed showed a preference for Leu in P2’ position (Figure 3B and S9B), similar to plant legumains.^27^ This activity also confirmed the structural integrity of the S2’ pocket in this mutant construct. Interestingly, the preference for Leu in P2’ was especially prominent for peptide substrates harboring aspartic acid at the P1 position (Figure 3D,E). Furthermore, we found that the V155G-D160Y mutant had significantly higher protease and transpeptidase activity toward the SFTI-GL substrate as evidenced by complete precursor turnover and approximately 60% cyclic product formed with a cyclization-to-hydrolysis ratio of 1.3:1 (Figure 3F). While the turnover of the SFTI(N14)-GL peptide was not reduced by 60% as we observed for the AAN-AMC fluorescence substrate (Figure S9C), the SFTI-GL peptide was hydrolyzed even better and the formation of cyclic product was increased in both cases, in agreement with increased substrate affinity.

Engineering the S2’ site by introducing a V155G-D160Y double mutant not only improved the affinity for prime-side ligase substrates but also increased the specificity of the prime peptidase substrates at the P2’ position (Figure 3B and S9B). In agreement with previous reports, this finding indicates that peptidase and ligase substrates share the identical prime (and obviously non-prime) recognition sites.^34−36^ As a consequence, the ligation reaction must follow a ping-pong mechanism, where the prime side peptidase product must first be released from the enzyme before the prime ligase educt can access and react with the acyl-enzyme intermediate.^37^

Knowing that the prime side interaction is critical for transpeptidation to proceed, we hypothesized that conversely the transpeptidation reaction could be boosted by increasing the concentration of the prime side substrate relative to the non-prime substrate. To test this hypothesis, the SFTI-derived peptides could not be used, as they harbor non-prime and prime side peptide on the same molecule. Therefore, in a next step, we expanded our transpeptidation/ligation assay to linear peptides. To reduce the complexity caused by sequence variations, we used simple Ala-Ala-Asn(-Ala) (AAN(A)) and Gly-Gly (GG) peptides, where AAN(A) served as the non-prime substrate and GG as the prime side substrate. When we coincubated these peptides together with legumain in a 1:1 molar ratio, we did not observe formation of an AANGG transpeptidation or ligation product (Figure S10A). However, when we increased the concentration of the prime side GG peptide by 20-fold, to mimic the effect of the SFTI precursor peptide, where the concentration of the prime side substrate is locally high, we observed the product AANGG for both AAN and AANA precursor peptides. However, the product formed was relatively low, probably because of re-cleavage of AANGG by legumain. Re-cleavage was not observed in time-series experiments for cyclic SFTI (Figure S10D), where the product was conformationally stabilized due to P2-Pro isomerization within the cyclic structure.^26^

The structure of legumain in complex with human cystatin E (pdb 4n6o) suggests that a substrate’s P1’ residue side chain must be in close proximity to the catalytic Cys189.^8^ Following this idea, we hypothesized that a Cys in P1’ position could help to increase the local concentration of the prime side substrate by disulfide formation with the catalytic Cys189. To test this hypothesis, we incubated legumain together with a Cys-Ile-Pro (CIP) peptide. As expected, we observed significant inhibition of legumain’s protease activity (Figure S10B). Inhibition was reversible when DTT was added. Additionally, we observed covalent modification of Cys189 using mass spectrometry, further confirming the covalent CIP binding. Even more interesting, we observed the formation of the AANCIP product when legumain was incubated with AANA or AAN and CIP even in a 1:1 molar ratio (Figure S10C). To test whether this positive effect was really due to disulfide formation with the catalytic cysteine residue rather than non-covalent interactions to the Ile-Pro sequence, we repeated the experiment using a Gly-Ile-Pro (GIP) control peptide. Indeed, we found less ligation product formed when the GIP peptide was used as a prime side substrate compared with the CIP peptide.

Prime side substrate affinity is a limiting factor for ligation but less so for proteolysis as protease substrates bind via prime and non-prime sites simultaneously. We therefore propose that increased prime side affinity will favor transpeptidase over protease activity by (i) increasing the residence time of the prime side ligation substrate and consequently (ii) decreasing the residence time of the catalytic water that would otherwise dissolve the covalent thioester intermediate and thereby complete the hydrolysis reaction. Introducing a plant-legumain-like S2’ pocket will have a positive effect especially for substrates harboring hydrophobic P2’ residues, in particular Leu in P2’ position. Alternatively we could show that a missing prime side pocket can be compensated by using an excess of the prime side substrate or by using Cys as P1’ ligase substrate. We would like to emphasize that while the prime recognition site of human legumain has only limited affinity and activity toward hydrophobic P2’ substrates, it may have high selectivity and ligase activity toward other prime-side substrates with yet undetermined amino acid preference. Analysis of electrostatic surface potentials suggest that a positively charged amino acid at position P2’ may have favorable affinity due to electrostatic interactions.

### ACP Is Ligase and Transpeptidase

**Figure 4:**
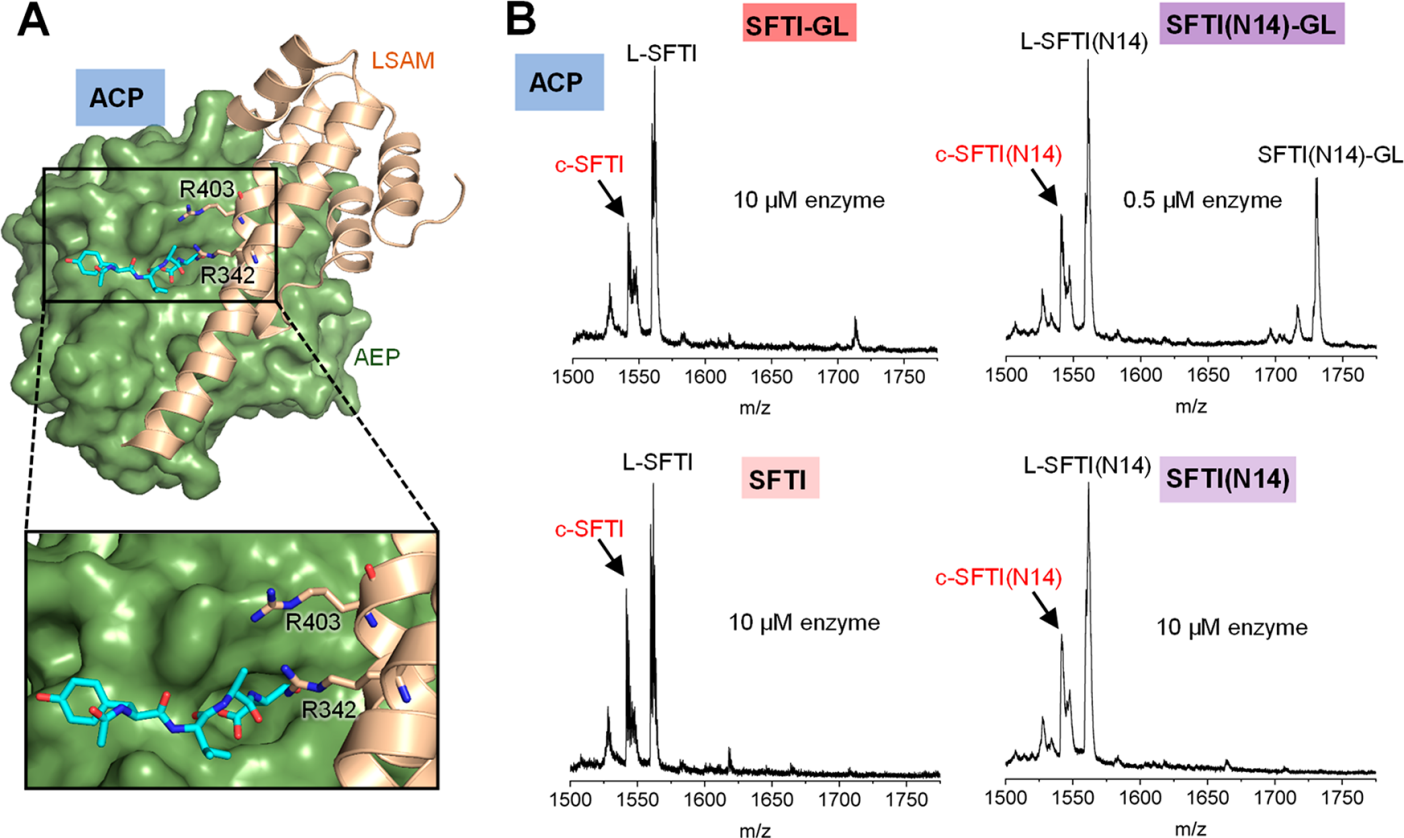
ACP harbors transpeptidase and ligase activity. (A) Structure model of ACP based on the crystal structures of human prolegumain (4fgu) and active AEP bound to the YVAD-cmk inhibitor (4aw9). The active site is indicated by the YVAD-cmk inhibitor, shown in blue sticks, the double arginine motif (R342 and R403) is shown in orange sticks. (B) SFTI-GL and SFTI(N14)-GL cyclization assay using the ACP activation intermediate.

The transpeptidase and ligase activities of human legumain essentially rely on near-neutral pH conditions. However, the stability of legumain is low at pH > 6.0 due to an electrostatic stability switch (ESS) that is encoded by an unbalanced negative surface potential surrounding the active site.^4^ Previously, we found a two-chain intermediate activation state where the activation peptide is removed, but the LSAM domain remains bound to the AEP domain.^4^ In this state, the non-prime substrate binding sites are accessible and allow for ACP (Asparaginyl CarboxyPeptidase) activity (Figure 4A). Importantly, the ACP state is stable at neutral pH, which prompted us to test if ACP would be a superior transpeptidase. Indeed, upon incubation of ACP with the SFTI-GL and SFTI(N14)-GL precursor peptides, we observed the formation of cyclic SFTI and SFTI(N14) products (Figure 4B). In agreement with the better pH-stability of ACP at pH 6.0, and contrasting AEP (Figure 1D), we observed complete turnover of the SFTI-GL precursor peptide, accompanied by about 25% cyclic product formation (cyclization-to-hydrolysis ratio 0.4:1). While we did not observe ligation mediated cyclization of the linear L-SFTI and L-SFTI(N14) precursor peptides by AEP (Figure S1B), ACP resulted in approximately 40% product formation when L-SFTI was used and approximately 30% product formation when L-SFTI(N14) was used with cyclization-to-hydrolysis ratios of 0.4:1 and 0.6:1, respectively (Figure 4B). To exclude that the ligase activity was contributed by the dissociation of the LSAM domain, we subjected the reaction to size exclusion chromatography (Figure S11). Importantly, the LSAM domain did not dissociate off the AEP domain upon incubation of ACP with the SFTI(N14)-GL substrate. Together, these results show that AEP and ACP differ in their catalytic activity not only when it comes to protease activity but also even more for ligase and transpeptidase activity. ACP is stable at neutral pH and therefore allows compensation for low-affinity P1-Asp substrates by increasing the incubation time/half-life. A conserved double arginine motif (Arg342 and Arg403), which we previously identified to be critical for the carboxypeptidase activity of legumain, may also assist the binding of non-prime ligase substrates (Figure 4A). Alternatively, the double arginine motif may serve as a positively charged anchor that attracts a short prime substrate’s C-terminus or possible carboxylate side chains, thereby increasing its affinity and facilitating the ligase activity of legumain. Also, the double arginine motif may favor the uncharged form of the N-terminus of the prime side ligase substrate and thereby foster transpeptidation.

### Charge Neutralization Leads to Conformational Stabilization of Legumain

**Figure 5:**
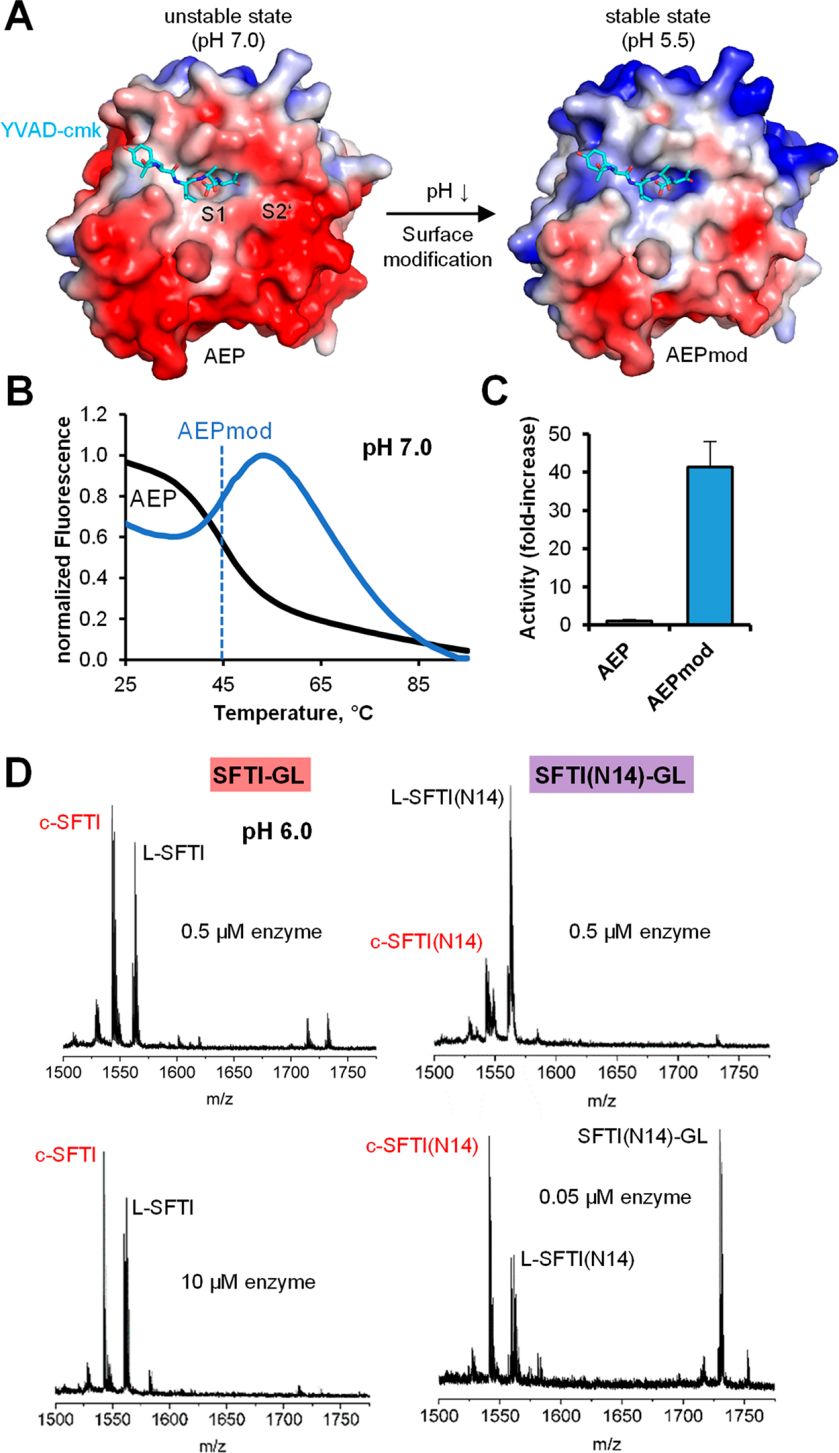
Legumain is pH-stabilized by surface modification. (A) Electrostatic surface potential of legumain calculated at pH 7.0 and pH 5.5 and visualized at ±5 e/*kT*. Negatively charged areas are shown in red, positively charged in blue and hydrophobic in gray. (B) Melting curves of wild-type legumain (black) and after modification with ethanolamine (AEPmod, blue) determined at pH 7.0 using differential scanning fluorimetry. (C) Activity of legumain and AEPmod toward the AAN-AMC substrate at pH 7.0. (D) Transpeptidase activity of AEPmod toward SFTI-GL and SFTI(N14)-GL peptides at indicated enzyme concentrations.

In a next step, we were then wondering if we could mimic the charge-neutralization effect of the LSAM domain by chemical modification of the ESS on AEP. Negative surface charges, as found at the ESS surrounding the legumain active site, are primarily encoded by amino acids harboring carboxyl groups in their side chain, that is, Asp and Glu (Figure 5A). To specifically neutralize these charged groups, we employed chemical surface modification. Since the surface surrounding the active site is not accessible in prolegumain, we carried out the modification with the isolated AEP domain. Specifically, we activated carboxylates by ethyl(dimethylaminopropyl) carbodiimide/*N*-hydroxysuccinimide (EDC/NHS), which were then covalently modified by reaction with ethanolamine. Attachment of ethanolamine to Asp or Glu eliminates the charge of these residues. After modification, we tested the thermal stability of AEPmod and indeed found that it was significantly increased at pH 7.0 as compared with unmodified wild-type legumain (Figure 5B), while there was expectedly little effect at pH 4.0 (Figure S12A). Even more important, we also observed a significant increase in enzymatic activity at pH 7.0 (Figures 5C and S12B). While wild-type legumain is nearly inactive at neutral pH, AEPmod showed an approximately 40-fold increase in activity toward the AAN-AMC substrate. To test the effect of modification on substrate specificity, we carried out PICS experiments (Figure S12C). Like wild-type legumain, the chemically modified legumain (AEPmod) showed the same strong preference for Asn in P1 position, independent of pH, and increased cleavage with Asp in P1 at very acidic pH (4.0).

In a next step, we tested the effect of surface modification on the transpeptidase activity of human legumain. When we used the P1-Asp containing SFTI-GL precursor peptide, we observed complete turnover of the precursor peptide into L-SFTI and c-SFTI even at 0.5 μM enzyme concentration, where the unmodified AEP was not able to catalyze cyclization (Figures 5D and 1C). Cyclic and linear products were observed in about a 1.2:1 ratio. Similarly, using the P1-Asn SFTI variant, we also observed an increase in cyclic product formation with approximately 25% and 40% cyclic product formed at 0.5 and 0.05 μM enzyme concentration, respectively (Figure 5D). However, the increase in precursor processing was not as pronounced as for the P1-Asp substrate. Furthermore, we also tested whether transpeptidation would work better at pH 6.5 using the pH-stabilized legumain (Figure S12D). Indeed, we observed an increase in precursor turnover and cyclic product formation for the P1-Asn variant (30% product formed). However, no cyclic product was generated when the P1-Asp variant was used. This is in excellent agreement with our PICS data, showing that the preference for Asp is decreasing with increasing pH.

Many proteins have unbalanced charged surface segments, limiting their function to the presence of balancing cofactors or a stabilizing environment, as exemplified by lysosomal proteases. We expect that chemical surface modification strategies such as amidation or esterification, as here introduced in legumain, may also stabilize other lysosomal proteases with high surface potential and render them stable in neutral pH environments.

### pH-Stable Legumain-Like Proteases Have Transpeptidase/Ligase Activity

**Figure 6:**
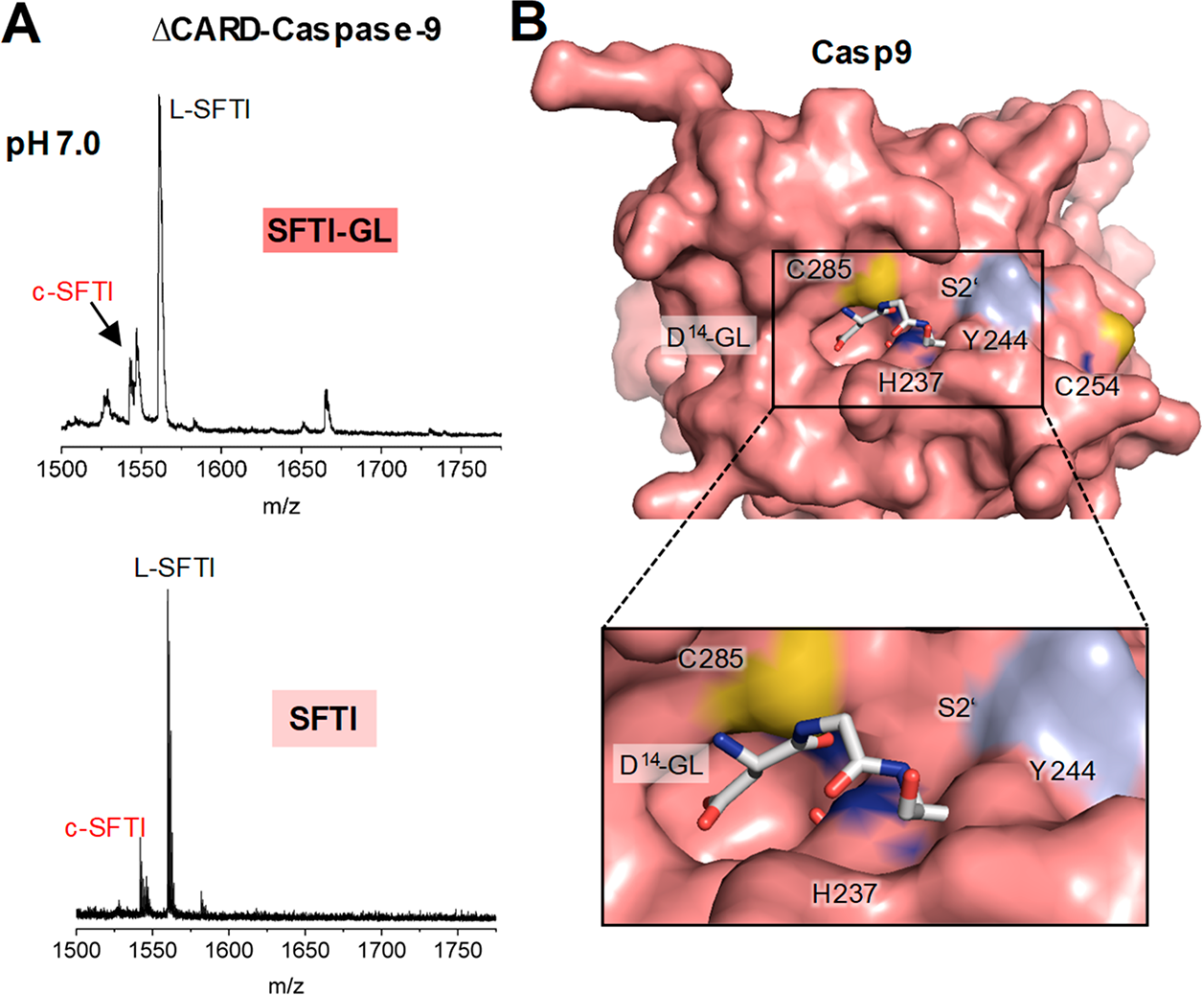
Caspase-9 has protease, ligase and transpeptidase activity. (A) Cyclisation of the SFTI-GL and SFTI precursor peptides was investigated at pH 7.0 and 10 μM enzyme concentration. (B) Surface representation of human caspase-9 (pdb 1jxq) showing the active site in complex with a modeled Asp-Gly-Leu peptide (P1-P1’-P2’; white sticks). Binding of the peptide was modeled on the basis of the structure of legumain in complex with cystatin E (4n6o; see Figure 3).

Observing that fold-stability is a critical factor for legumain transpeptidase activity, we were speculating whether legumain-like proteins that are intrinsically stable at neutral pH, would be even better ligases. Human caspases have a legumain-like fold, specificity for Asp in P1 and are stable at neutral pH. Therefore, we hypothesized that caspases might be able to cyclize the SFTI-GL precursor peptide. Indeed, we did observe cleavage and cyclization of this peptide by ΔCARD-caspase-9, which we used as a model enzyme (Figure 6A). Interestingly, caspase-9 could even cyclize the L-SFTI peptide, lacking the GL on the prime side, showing that it can catalyze both transpeptidation and ligation reactions (Figure 6A), although, compared with human legumain, the cyclization efficiency was low (approximately 10–20%). To exclude that the mass corresponding to the cyclic product was not due to the spontaneous formation of a cyclic anhydride at the C-terminal Asp14,^38^ we set up similar assays using N-acetylated SFTI control peptide and SFTI(N14)-GL: the former bears a blocked N-terminus that cannot undergo cyclization, the latter does not have Asp in P1 position and should therefore not serve as a substrate to caspase-9. Both control peptides were resistant to caspase-9-catalyzed ligation (Figure S13).

Ligase activity was previously observed also in gingipains.^39^ It is tempting to speculate that also other members of the clan CD proteases harbor ligase activity. Because of their highly different substrate preferences and subtle differences in active site architecture, we expect the activity of specific clan CD enzymes to be different. Caspase-9 as an example has a rather narrow prime side and will therefore require substrates that present small residues in P1’ and P2’ positions (Figure 6B). Furthermore, it lacks Snn147 and Ser215 (Figure S7), which we identified as critical regulators of legumain transpeptidase and ligase activities.

## Conclusion

**Figure 7:**
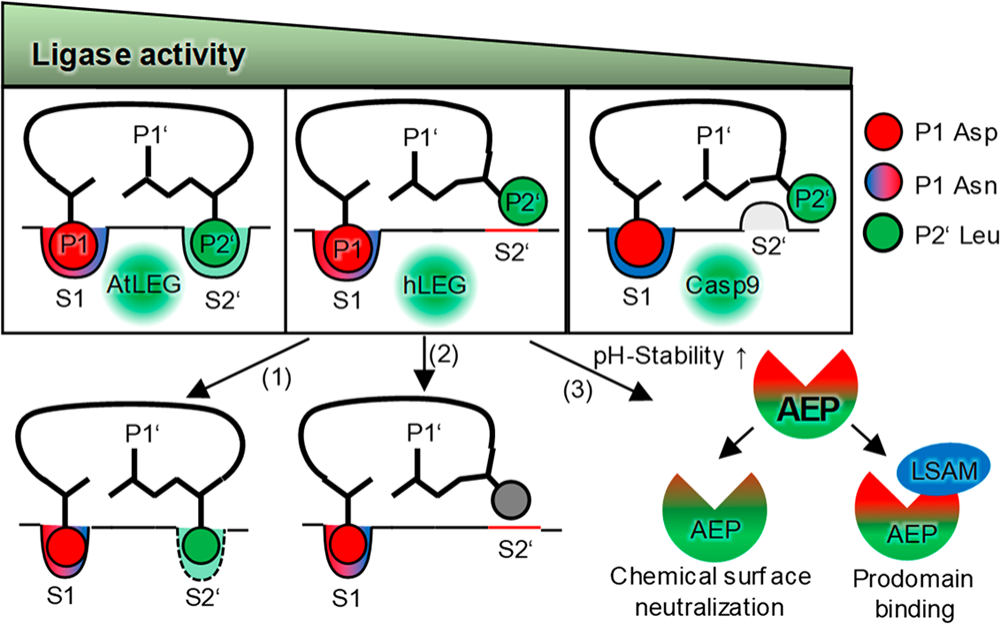
Regulation of legumain activities. Peptide cyclization works most efficiently with plant legumains, followed by human legumain, followed by caspase-9. The ligase activity of human legumain can be improved by (1) engineering an S2’ pocket, (2) by optimizing the substrate sequence for improved affinity to the S2’ site, and (3) by improving legumain’s pH-stability at near-neutral pH.

Within this study, we could for the first time show that mammalian legumain harbors peptide ligase and transpeptidase activities by which it can catalyze peptide cyclization. Ligase and transpeptidase activities both require near-neutral pH, which is however not compatible with legumain’s fold stability. To turn legumain into an even better transpeptidase, we developed two complementary strategies, which rely on (i) increasing fold stability and (ii) improving prime-side substrate affinity (Figure 7). Increasing the stability of legumain at near-neutral pH is beneficial not only for transpeptidase reactions but also for its protease function. Especially, substrates harboring Asp in P1 position will benefit from legumain’s extended lifetime, thereby compensating for their high *K*_M_ at near-neutral pH. Both the transpeptidase activity combined with the strict substrate preference of legumain make it an attractive target for biotechnological applications. However, most of these applications require (near) neutral pH conditions. Therefore, the herein described ACP and chemically stabilized variants of legumain represent important new tools for example in proteomics experiments or protein modification assays. We recently showed that legumain is a valuable alternative and complement to trypsin in mass spectrometry-based experiments. Nonetheless, the usability of the wild type protease is limited because of the opposing pH-dependence of its protease activity, with an optimum at pH 5.5, and the solubility of the generated peptides, which is decreasing with lowering pH.^30^ Therefore, stabilized legumain variants will further improve its applicability in proteomics experiments. Also in the field of site-specific bioconjugation and native chemical ligation legumain offers an attractive alternative and complement in the tool box which is short of useful enzymes such as subtiligase.^40^ In particular its ability to catalyze ligation reactions without the prerequisite of thioester formation makes ACP, the two-chain form of legumain, an indispensable expansion of the ligation toolbox, enabling site-specific labeling of proteins or peptides at free and unmodified C-terminal Asn residues.

Additionally, we found that transpeptidase and ligase activity are not strictly limited to legumain but are an intrinsic feature of structurally related enzymes like caspases, although with lower efficiency. Because of their different specificity in P1 position (Asp, Asn, Lys, Arg), together the clan CD proteases provide a large repertoire of enzymes with application for various substrates. Recently, the structurally related gingipains were shown to have transpeptidase activity toward human hemoglobin, which may potentially result in autoimmune reactions.^39^ Interestingly, the proteaseome, which is the major protease responsible for the production of peptides presented on MHC class I, was also shown to produce spliced antigenic peptides through a transpeptidation reaction.^41^ Similar to legumain, the proteasome employs the acyl-enzyme intermediate, which is subjected to a nucleophilic attack by the free amino group of a prime side peptide. Spliced peptides of, for example, the FG-5 protein or gp100 were shown to be presented by melanoma cells. Similarly, the lysosomal cysteine protease cathepsin L was shown to generate chimeric fusion epitopes via transpeptidation of peptides derived from chromogranin A and islet amyloid polypeptide with high antigenicity for diabetogenic CD4 T cells.^42^

Furthermore, we also learned new lessons on the catalytic mechanism of legumain. The analysis of the active site of legumain in complex with a ligase substrate revealed that the catalytic Cys189 exists in two orientations that will favor either hydrolysis/transpeptidation or ligation. We identified Ser215, which is hydrogen bonding to the protease orientation of Cys189, as a previously unknown regulator of its protease/transpeptidase activity (Figure 2F).

Taken together, we could show that human legumain harbors peptide transpeptidase and ligase activities prevailing at near-neutral pH. Although numerous studies document the relevance of extra-lysosomal legumain for pathologic disorders, its ligase function at these locations was not studied so far. Many ligation products, and consequently substrates, remain undiscovered, because they are challenging to find ab initio by mass spectrometry-based approaches. However, it is tempting to speculate that the ligase and transpeptidase activities of human AEP and especially ACP forms may play a so far overlooked role in tumor progression, neurodegenerative disorders, and antigen processing by cross-linking specific protein or peptide targets.

## Supporting Information Available

Experimental procedures and supporting figures (PDF)
