# Supplementary material for: The Peptide Ligase Activity of Human Legumain Depends on Fold Stabilization and Balanced Substrate Affinities
Source: ACS Catal. 2021 Sep 10;11(19):11885–96. doi: 10.1021/acscatal.1c02057 (PMC8491156; doi:10.1021/acscatal.1c02057)
Supplement: Supplementary file 1 — cs1c02057_si_001.pdf [file cs1c02057_si_001.pdf]

Supporting Information

The peptide ligase activity of human legumain depends on fold stabilisation and balanced substrate affinities

Elfriede Dall[†*], Vesna Stanojlovic[†], Fatih Demir[§#], Peter Briza[†], Sven O. Dahms[†], Pitter F. Huesgen[§‖⊥], Chiara Cabrele[†], and Hans Brandstetter[†*]

Affiliations:
[†]Department of Biosciences, University of Salzburg, 5020 Salzburg, Austria.
[§]Central Institute for Engineering, Electronics and Analytics, ZEA-3, Forschungszentrum Jülich, 52428 Jülich, Germany.
[#]present address: Department of Biomedicine, Aarhus University, 8000 Aarhus C, Denmark
[‖]CECAD, Medical Faculty and University Hospital, University of Cologne, 50931 Cologne, Germany
[⊥]Institute for Biochemistry, Faculty of Mathematics and Natural Sciences, University of Cologne, 50674 Cologne, Germany
[*]To whom correspondence should be addressed.

Corresponding Authors:
Elfriede Dall, Department of Biosciences, University of Salzburg, 5020 Salzburg, Austria; Email: elfriede.dall@plus.ac.at
Hans Brandstetter, Department of Biosciences, University of Salzburg, 5020 Salzburg, Austria; Email: hans.brandstetter@plus.ac.at

EXPERIMENTAL PROCEDURES

Protein preparation

Human legumain was expressed, purified and activated as described previously [1]. Point mutants were generated using round-the-horn site directed mutagenesis, which is based on the inverse PCR method, as described earlier [2, 3]. Caspase 9 was cloned into the pet22b vector using XbaI and XhoI. The expression construct carried the catalytic domain of human caspase 9 and an C-terminal His_6-tag. ΔCARD-caspase-9 was expressed in BL21(DE3) cells as described elsewhere [4]. Briefly, BL21(DE3) cells containing the ΔCARD-caspase-9 expression construct were grown in LB medium at 37 °C till an OD600 of 0.5 – 0.7 was reached. Subsequently cells were transferred to 25 °C and expression was induced upon addition of 0.4 mM IPTG. After 4 hours of expression, cells were harvested by centrifugation at 4000 rpm, 4 °C for 10 min. The pellet was resuspended in lysis buffer composed of 50 mM Hepes pH 7.5 and 100 mM NaCl. Cells were lysed by sonication (45 seconds, 40% power, 50% cycle, 3 times) and the supernatant containing soluble caspase-9 was harvested by centrifugation (17500 g, 15 min, 4 °C). The cleared supernatant was incubated with Ni^{2+}-resin for 15 min at 4 °C. After protein binding, the beads were washed with 5 column volumes of lysis buffer and one column volume of lysis buffer supplemented with 5 mM, 10 mM and 15 mM imidazole respectively. Bound protein was eluted with lysis buffer supplemented with 250 mM imidazole. Elution fractions were concentrated using Amicon ultra centrifugal filter units (cutoff: 3 – 5 kDa) and subjected to size exclusion chromatography using an S75 column pre-equilibrated in 20 mM Hepes pH 7.5 and 100 mM NaCl. The final protein migrated as 3 distinct bands on SDS-PAGE, corresponding to the p19, p18 and p12 subdomains. Fractions containing pure caspase-9 were pooled, concentrated, and frozen in aliquots at -20 °C.

Fluorescent activity assay

Legumain protease activity was tested using the Z-Ala-Ala-Asn-7-amino-4-methylcoumarin (AAN-AMC; Bachem) fluorescent peptide at an enzyme concentration of 4 nM, if not stated differently. Activity of wild-type and different mutants was assayed at pH 5.5, unless otherwise stated, in assay buffer composed of 50 mM citric acid, 100 mM NaCl, 0.05% Tween-20, and 50 µM AAN-AMC. To test the pH-stability of legumain, activity was measured in buffer composed of 50 mM Hepes pH 7.0, 100 mM NaCl, 0.05% Tween-20. Increase in fluorescence signal was monitored at 460 nm after excitation at 380 nm in an Infinite M200 Plate Reader (Tecan) at 37 °C. Activity of caspase-9 was assayed in a buffer composed of 0.5 M Na_3citrate, 50 mM Hepes pH 7.5, 100 mM NaCl, and 0.05% Tween-20 supplemented with 100 µM Z-Val-Ala-Asp-AMC (VAD-AMC; Bachem) substrate. Activity was measured at 37 °C at an enzyme concentration of 1 µM.

Chemical surface modification

Active legumain was buffer exchanged to 100 mM MES pH 6.0 and 100 mM NaCl at a final protein concentration of 0.05 mg/ml. EDC and NHS stock solutions were prepared freshly by dissolving appropriate amounts of powder in ddH_2O. Concentrations of the stock solutions were 400 mM for EDC and 100 mM for NHS. EDC and NHS were mixed in a 1 : 1 ratio and added to legumain at a final concentration of 80 mM and 20 mM respectively. The reaction was incubated for 10 min. at 22 °C. After 10 min., ethanolamine was added at a final concentration of 166 mM and the reaction was incubated for another 10 min at 22 °C. A 1 M ethanolamine stock solution was used which was prepared by diluting the 16.5 M stock solution with 1 M citric acid and adjusting pH to 6.2. After completion of the reaction, the reaction buffer was exchanged to 20 mM citric acid pH 4.0 and 100 mM NaCl using a NAP-5^{TM} column and the protein was further concentrated using a vivaspin concentrator (Satorius; MW cutoff: 10 kDa). Additionally, a control

reaction was prepared in parallel, that was treated essentially the same, but supplemented with ddH_2O instead of EDC/NHS and ethanolamine.

Peptide synthesis

SFTI-derived precursor peptides (SFTI-GL, SFTI, SFTI(N14)-GL, SFTI(N14), and N-acetylated SFTI) were synthesized as described previously [5]. AAN, AANA, CIP and GIP peptides which were used for the ligation assays were purchased from JPT (Berlin). GG was purchased from Sigma Aldrich (Darmstadt). Ac-GSN was synthesized by Fmoc-chemistry using a H-Asn(trityl)-2-chlorotrityl resin (Merck Schuchardt OHG, Hohenbrunn, Germany) and the methods reported previously (MS for $C_{11}H_{19}N_4O_7^+$: 319.29 Da; MS found: 319.35 Da) [5].

Cyclisation assays

SFTI-derived peptides were synthesized and analyzed as described previously [5]. Subsequently, cyclization experiments were carried out using 500 µM of the respective linear peptide and 0.05 µM, 0.5 µM or 10 µM legumain variant in a buffer composed of 100 mM NaCl and 50 mM citric acid pH 4.0 or pH 6.0 or 50 mM Bis-Tris pH 6.5. Reactions were incubated at 37 °C for 16 hours. Subsequently the reactions were desalted using C18 StageTips and analyzed by MALDI-TOF-MS (Autoflex, Bruker Daltonics, matrix: α-cyano-4-hydroxycin-namic acid). Reactivity of caspase-9 towards different SFTI-derived peptides was assayed in a buffer composed of 100 mM NaCl, 50 mM Hepes pH 7 and 0.75 M Na_3citrate at an enzyme concentration of 10 µM and 0.5 mM of the respective peptide.

Selected samples were additionally subjected to HPLC-MS experiments. Samples were analyzed by nanoHPLC (Dionex Ultimate 3000, Thermo Fisher Scientific, Bremen, Germany) coupled via nano electrospray to a Q Exactive Orbitrap mass spectrometer (Thermo Fisher Scientific). Data

analysis and de novo sequencing was done with PEAKS Studio X (Bioinformatics Solutions, Waterloo, Canada).

ACP stability assay

To test whether the two-chain complex of legumain (ACP; Asparaginyl Carboxpeptidase), which is composed of the AEP domain and the LSAM domain, is stable in the presence of the SFTI(N14)-GL substrate, we subjected it to size exclusion experiments. Specifically, we incubated 10 µM ACP with 25 µM SFTI(N14)-GL peptide in a buffer composed of 50 mM citric acid pH 6.0 and 100 mM NaCl for 2 hours at 37 °C. Subsequently we loaded the reaction on a S75 size exclusion column preequilibrated in reaction buffer at pH 6.0. Peak fractions were analyzed by SDS-PAGE.

Covariance analysis

We used CoeViz (4) to do a pairwise coevolution analysis of amino acid residues. A multiple sequence alignment was generated using PSI-BLAST and coevolution scores were computed using different covariance metrics. We identified closely related amino acid residues after applying a ≥ 0.3 cutoff to χ^2 scores.

Thermal stability assay

The thermal stability of different legumain variants was assayed using differential scanning fluorimetry. The respective protein was diluted into assay buffer containing 100 mM NaCl and 100 mM Hepes pH 7.0 or citric acid pH 4.0 to a final concentration of 0.2 mg/ml and supplemented with 10x Sypro Orange Dye (Sigma). Thermal unfolding was measured in a 7500 Real Time PCR System (Applied Biosystems) after increasing temperature by 1 °C per min from 20 °C to 95 °C

and detecting fluorescence signal. Fluorescence data was normalized to peak values and melting curves were evaluated as described elsewhere [6].

PICS experiments

To test the substrate specificity of human legumain we carried out Proteomic Identification of protease Cleavage Sites (PICS) assays using peptide libraries generated from *Escherichia coli* BL21 proteome extracts as described previously [7-9]. The proteome was dissolved in 100 mM Hepes pH 7.5 and digested overnight with 22 µg/µl trypsin at 37 °C. The hereby generated peptide library (2 mg/ml) was incubated with human legumain variants (10 µg/ml) in assay buffer composed of 50 mM buffer substance (pH 4.0 and 5.5: citric acid; pH 6.5: MES) and 100 mM NaCl at 22 °C. Samples were taken after 20 min and 16 h of incubation. Protease treated samples were isotope-labeled with heavy formaldehyde ($^{13}CD_2O$) and control reactions with light formaldehyde (CH_2O) for 2 h and quenched with 100 mM Tris pH 8.0. Protease-treated and control samples were mixed and purified using C18 StageTips.

Desalted peptides were analyzed using a nano HPLC (Ultimate 3000 RSLC, Thermo, Dreieich, Germany) operated in two-column setup coupled to a high resolution Q-TOF mass spectrometer (ImpactII, Bruker, Bremen, Germany). MaxQuant v1.6.0.16 [10] was used to match spectra to peptides from the UniProt *E.coli* K12 proteome library (downloaded Nov 2015, 4313 entries) with appended MaxQuant standard contamination entries. Trypsin was set as semi-specific digestion enzyme (i.e. only one side of the peptide was required to match the trypsin specificity). Label multiplicity was set to two, considering light dimethylation (+28.0313 Da) and heavy dimethylation (+34.0631 Da) as peptide N-terminal and lysine labels. Carbamidomethylation of cysteine residues (+57.0215 Da) was set as fixed modification, methionine oxidation (+15.9949 Da) and protein N-terminal acetylation (+ 42.0106 Da) were considered as variable

modifications. PSM false discovery rate was set to 0.01. Identified peptides that showed at least a fourfold increase in intensity after protease treatment compared to the control treatment or were exclusively present in the protease-treated condition were considered as putative cleavage products. An in-house Perl script (https://sourceforge.net/projects/pincis/) was used to remove putative library peptides (trypsin specificity on both sides of the identified peptide) and to reconstruct the full cleavage windows from the identified cleavage products as described [7]. Aligned validated cleavage windows were visualized using the iceLogo software version 1.3.8 [11], displaying site-specific differential amino acid abundance compared to the *E.coli* K12 proteome as reference set (p-value 0.05). Mass spectrometry data have been deposited to the ProteomeXchange Consortium via the PRIDE (https://www.ebi.ac.uk/pride/archive/) (59) repository with the dataset identifier PXD025570.

Ligation of linear peptides

15 µM legumain were mixed with 2 mM Ala-Ala-Asn (AAN) or Ala-Ala-Asn-Ala (AANA) peptide and 2 or 40 mM Gly-Gly (GG) peptide or 2 mM Cys-Ile-Pro (CIP) or Gly-Ile-Pro (GIP) peptide in a buffer composed of 50 mM citric acid pH 6.0 and 100 mM NaCl at 22 °C for 16 hours. Formation of ligated product was measured via mass spectrometry experiments. When CIP was used as primed side substrate, samples were reduced/alkylated and analyzed by nanoHPLC (Dionex Ultimate 3000, Thermo Fisher Scientific, Bremen, Germany) coupled via nano electrospray to a Q Exactive Orbitrap mass spectrometer (Thermo Fisher Scientific). When GG and GIP were used as primed side substrate, samples were directly infused into the Q Exactive mass spectrometer. Data analysis and de novo sequencing was done with PEAKS Studio X (Bioinformatics Solutions, Waterloo, Canada).

To confirm covalent modification of legumain with the CIP tripeptide, we incubated legumain with CIP as described above, and tested its activity towards the AAN-AMC substrate at pH 5.5. In a control reaction, we added DMSO instead of the CIP peptide.

Crystallization and structure solution

N263Q-legumain activated at pH 4.0 was crystallized in a condition composed of 22 % PEG 3350 and 0.1 M sodium acetate pH 4.5. 0.2 µl of the protein solution at 20 mg/ml were mixed with 0.2 µl precipitant at 293 K. Crystals appeared after 6 weeks. Crystals were soaked with 5 mM of the Ac-GSN peptide and harvested after 48 hours. For cryo protection in liquid nitrogen, crystals were soaked in crystallization buffer supplemented with 20% glycerol. X-ray data was collected at the ESRF (Grenoble) on beamline ID23-2 at 100 K. Crystals diffracted to a resolution of 2.0 Å. Data processing was performed using iMOSFLM and Aimless from the CCP4 program suite [12, 13]. PDB entry code 4awa was used as a search model for molecular replacement using PHASER [14]. Iterative cycles of model building in COOT followed by refinement in phenix.refine were carried out [15, 16]. The final structure was analyzed using PROCHECK and MolProbity and coordinates and structure factors were deposited with the PDB under the entry code 7O50. Molecular graphics were prepared with Pymol. Electrostatic surface potentials were prepared using APBS after assigning charges at pH 7.0 with Pdb2pqr.

SUPPORTING FIGURES

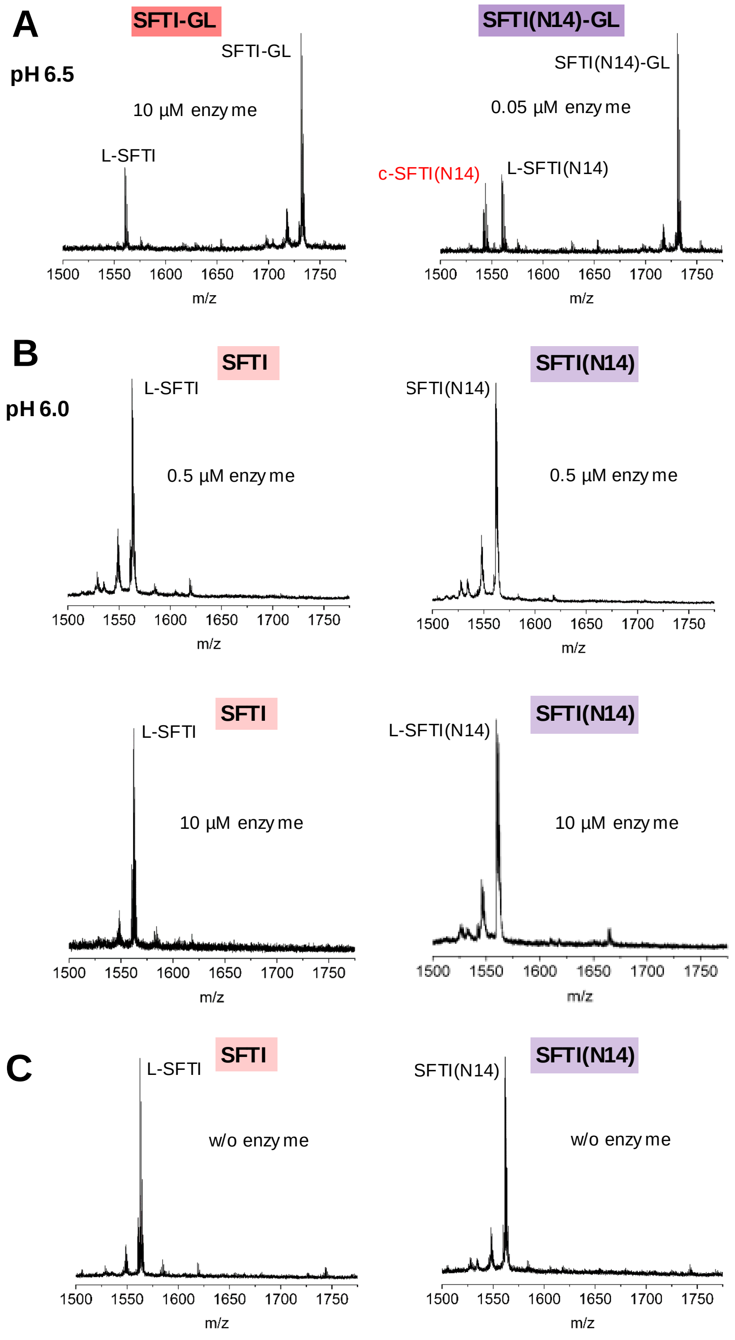

Figure S1. P1-Asn is the preferred legumain transpeptidase substrate. (A) Reactivity of legumain towards SFTI-GL and SFTI(N14)-GL peptides was assayed at pH 6.5. Both peptides were processed to the linear L-SFTI or L-SFTI(N14) to some extent, but cyclic product was only

observed with P1-Asn. (B) Legumain was not able to cyclise linear L-SFTI and L-SFTI(N14) peptides. (C) Control reactions without addition of legumain.

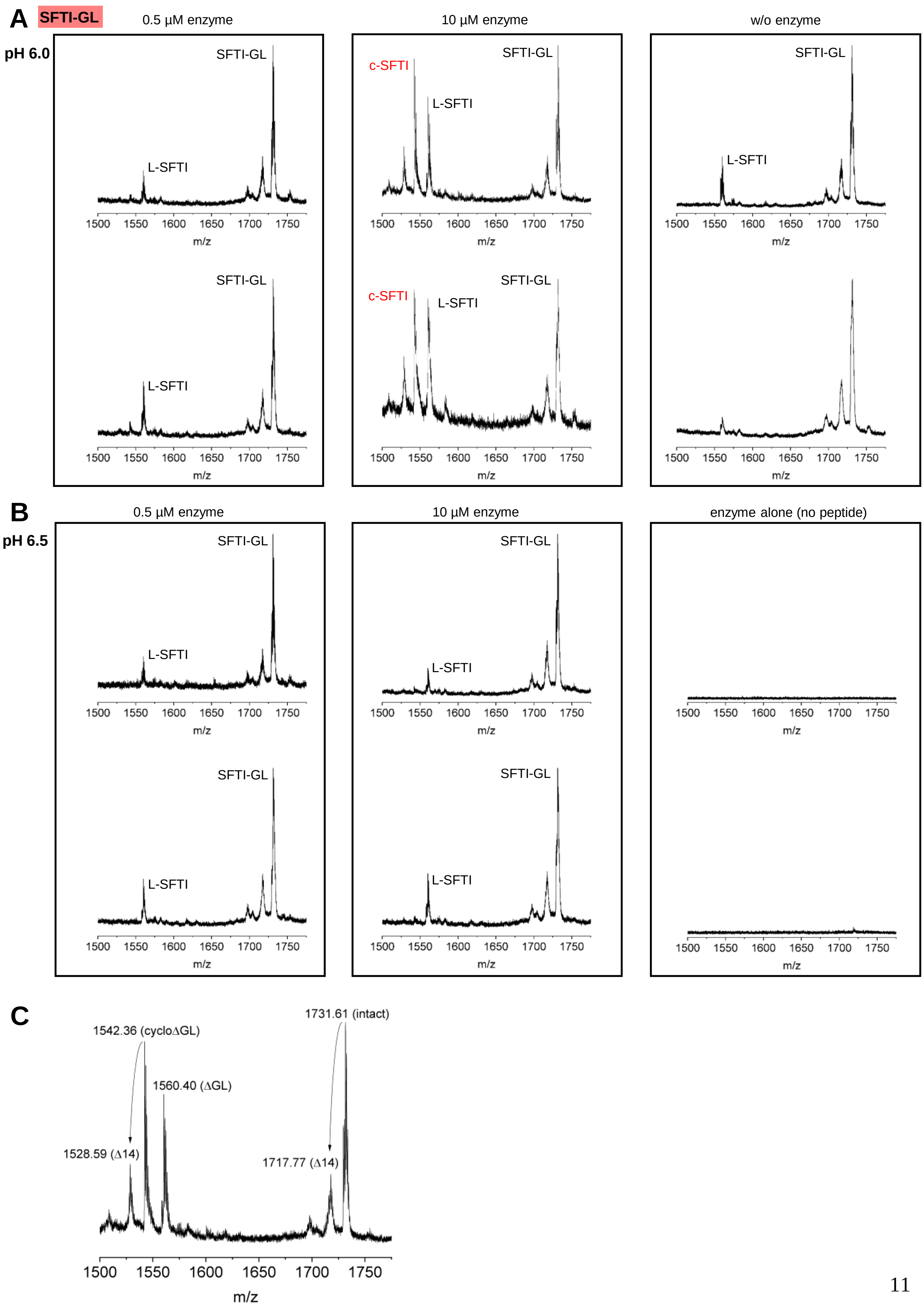
A
SFTI-GL
pH 6.0
0.5 µM enzyme
10 µM enzyme
w/o enzyme
SFTI-GL
L-SFTI
c-SFTI
m/z
B
pH 6.5
0.5 µM enzyme
10 µM enzyme
enzyme alone (no peptide)
C
1542.36 (cycloΔGL)
1731.61 (intact)
1560.40 (ΔGL)
1528.59 (Δ14)
1717.77 (Δ14)
1500 1550 1600 1650 1700 1750

Figure S2. Duplicate measurements confirm the reproducibility of the cyclisation experiments. (A) MS spectra of the SFTI-GL precursor peptide after incubation with 0.5 µM, 10 µM and 0 µM of legumain at pH 6.0. Cyclic c-SFTI product was only observed in the presence of 10 µM enzyme. (B) MS spectra of SFTI-GL precursor peptide after incubation with 0.5 µM or 10 µM enzyme at pH 6.5 and spectra of samples containing only the enzyme, but no peptide. No cyclic product was observed at pH 6.5. (C) MS spectrum of the SFTI-GL precursor peptide after incubation with 10 µM legumain at pH 6.0. The peak directly next to the SFTI-GL peak (intact, 1731.61 m/z) results from fragmentation induced demethylation (Δ14 Da, 1717.77 m/z), most likely after Thr4 or Leu16. The Peak directly left to cyclic c-SFTI (cycloΔGL; 1542.36 m/z) similarly corresponds to a fragmentation product (Δ14 Da, 1528.59 m/z).

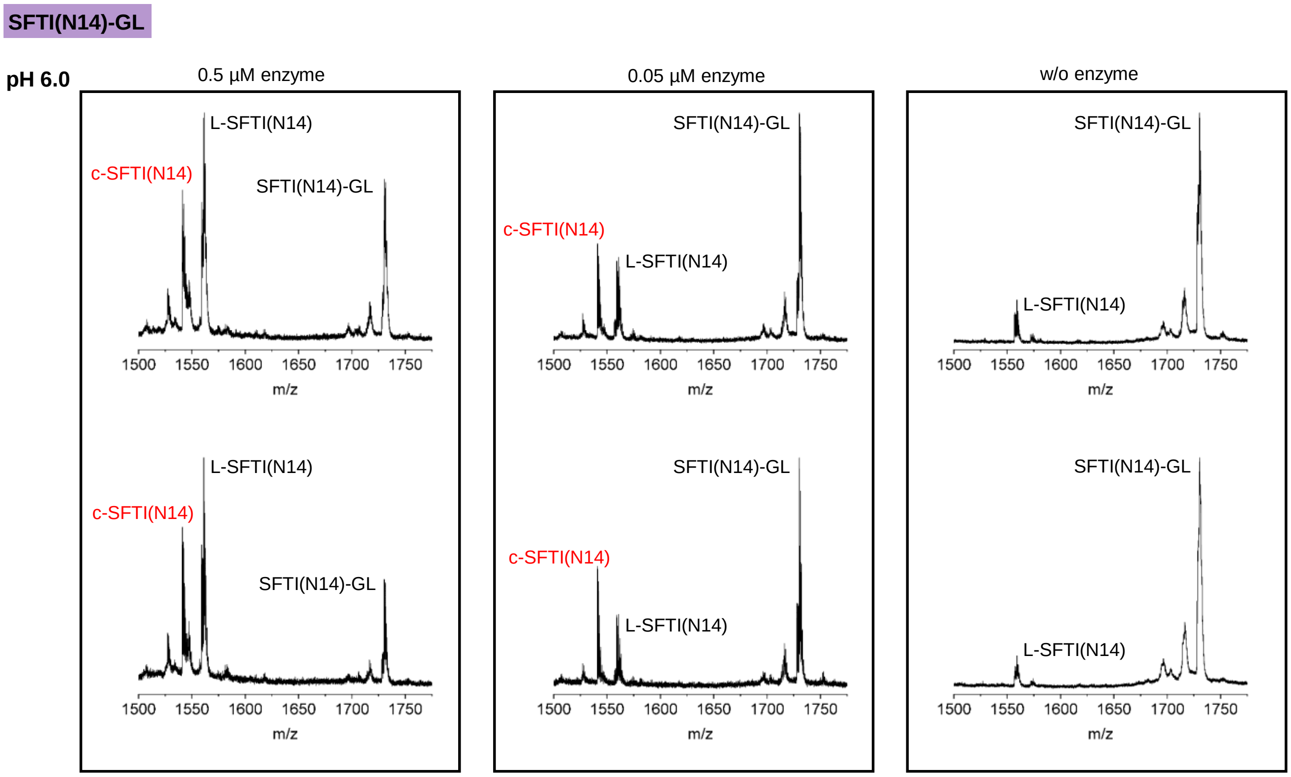

Figure S3. Duplicate measurements of the cyclisation experiments using the SFTI(N14)-GL precursor peptide. The SFTI(N14)-GL precursor peptide was incubated with legumain at 0.5 µM, 0.05 µM and 0 µM concentrations. Formation of the cyclic product c-SFTI(N14) was observed only in the presence of the enzyme.

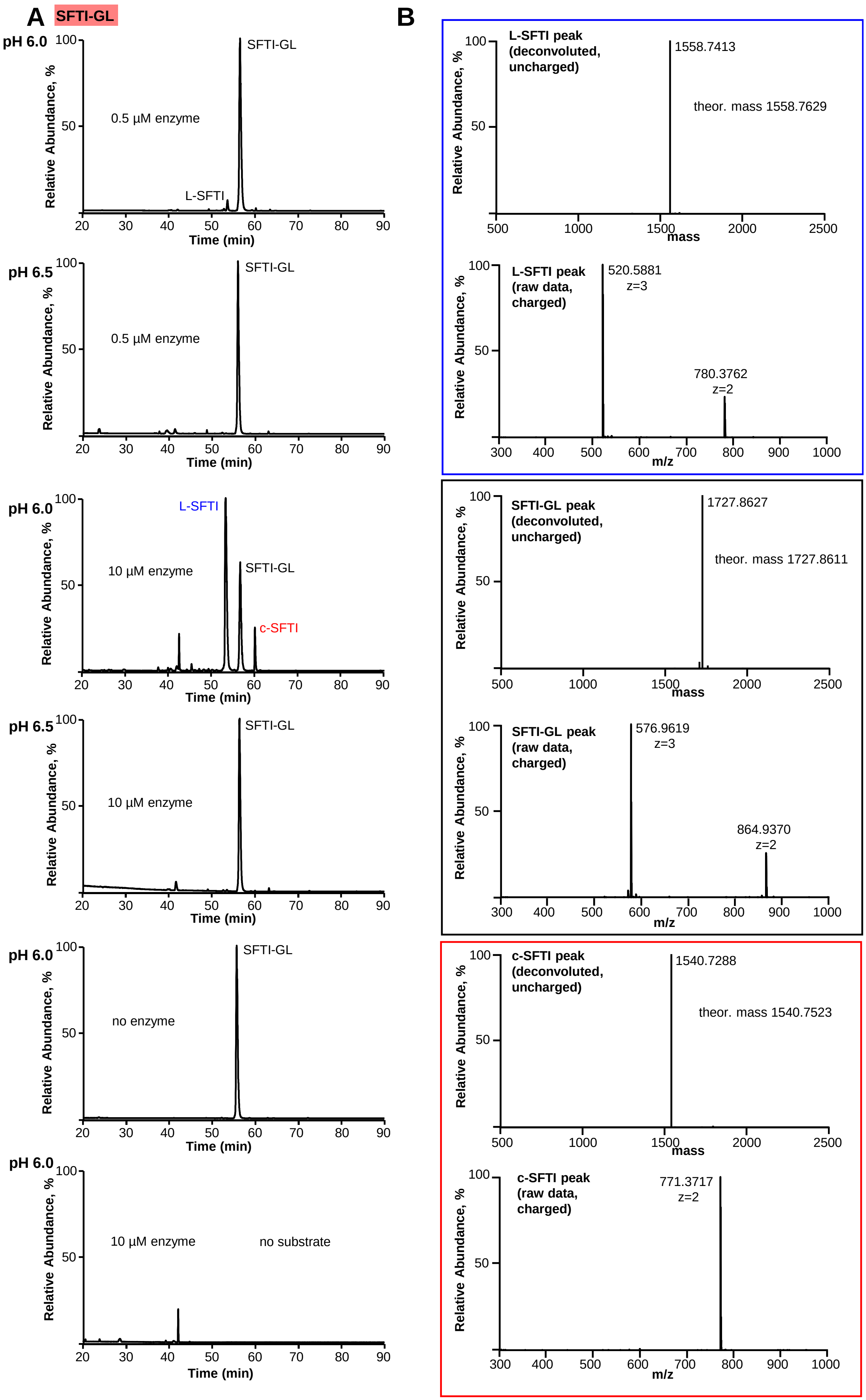
A
SFTI-GL
pH 6.0
0.5 µM enzyme
SFTI-GL
L-SFTI
pH 6.5
0.5 µM enzyme
SFTI-GL
pH 6.0
10 µM enzyme
L-SFTI
SFTI-GL
c-SFTI
pH 6.5
10 µM enzyme
SFTI-GL
pH 6.0
no enzyme
SFTI-GL
pH 6.0
10 µM enzyme
no substrate
Relative Abundance, %
Time (min)
B
L-SFTI peak (deconvoluted, uncharged)
1558.7413
theor. mass 1558.7629
L-SFTI peak (raw data, charged)
520.5881 z=3
780.3762 z=2
SFTI-GL peak (deconvoluted, uncharged)
1727.8627
theor. mass 1727.8611
SFTI-GL peak (raw data, charged)
576.9619 z=3
864.9370 z=2
c-SFTI peak (deconvoluted, uncharged)
1540.7288
theor. mass 1540.7523
c-SFTI peak (raw data, charged)
771.3717 z=2
mass
m/z

Figure S4. HPLC-MS experiments confirm cyclisation of the SFTI-GL precursor peptide by wild-type legumain at pH 6.0. (A) HPLC-MS chromatograms after incubation of the SFTI-GL precursor peptide with wild-type legumain at pH 6.0 and 6.5 and 0.5 µM and 10 µM enzyme concentration. Control experiments show the precursor peptide alone and the enzyme alone. (B) Mass spectra of the peaks corresponding to linear L-SFTI, SFTI-GL and cyclic c-SFTI after incubation at pH 6.0 and 10 µM enzyme concentration. The charged raw data show that c-SFTI lacks the z=3 state, consistent with the missing free N-terminus after cyclisation.

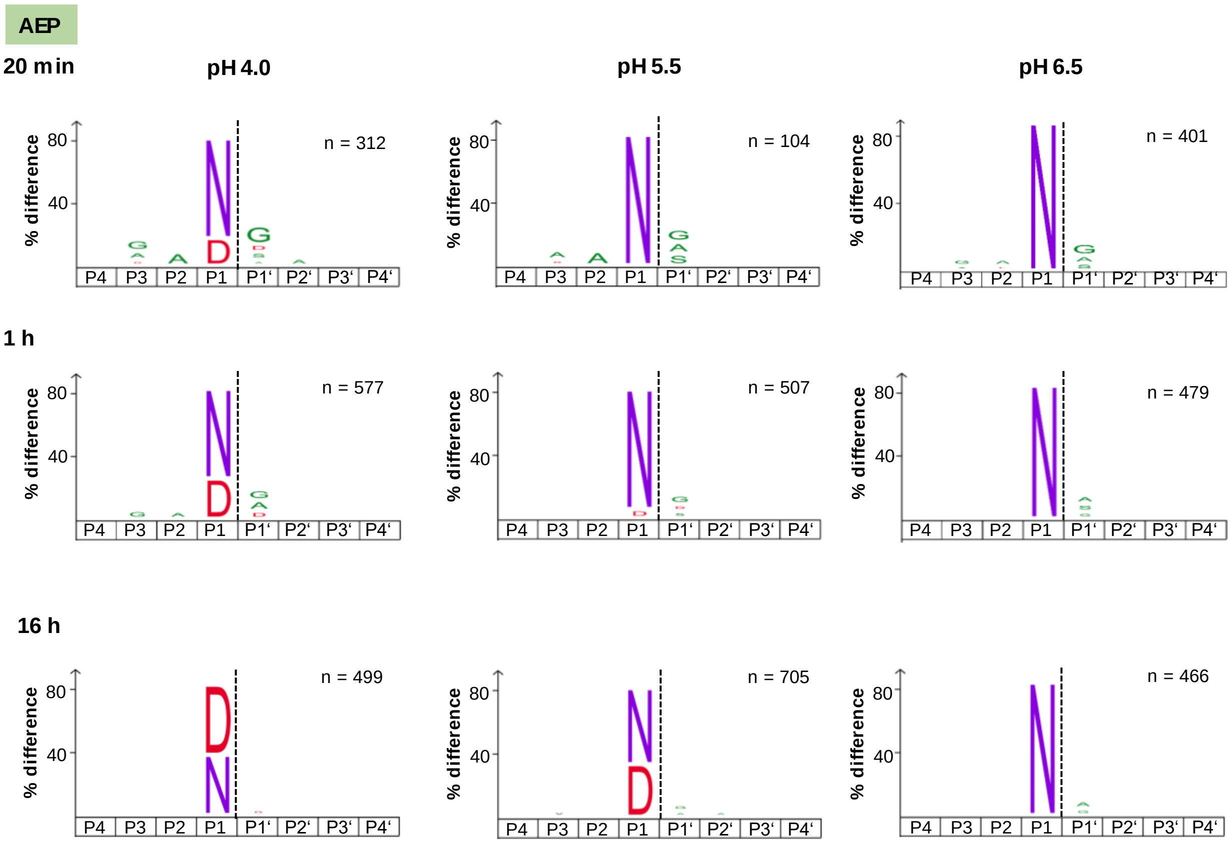

Figure S5. The substrate specificity of legumain is dependent on pH and time. IceLogos were prepared after 20 min, 1 hour and 16 hours incubation of the *E.coli* peptide library with legumain at pH 4.0, pH 5.5 and pH 6.5 (p = 0.05). Legumain shows a strict specificity for Asn at pH 6.5. Although Asp is tolerated at P1 position at pH 4.0, Asn is still the preferred substrate. Small hydrophilic residues are favourable at P1' position.

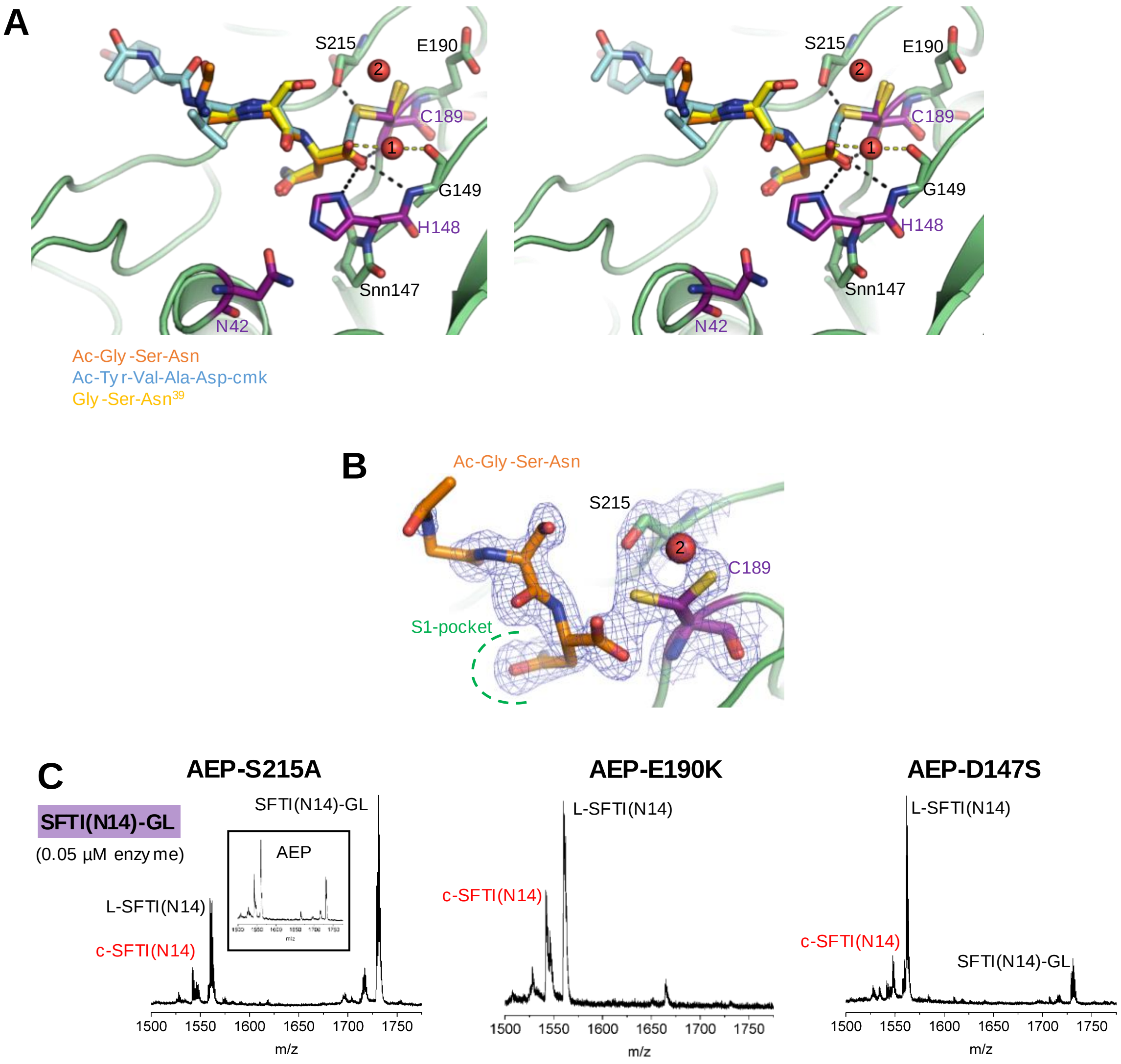

Figure S6. The Ac-Gly-Ser-Asn-tripeptide binds to the legumain active site in a substrate-like manner. (A) Stereo view on the active site of human legumain (green carbons) bound the GSN peptide (orange carbons). The Ac-YVAD-cmk inhibitor (blue carbons) was superimposed based on pdb 4aw9 and the Gly-Ser-Asn39 RCL segment (yellow) based on the crystal structure of legumain in complex with cystatin E (pdb 4n6o). Catalytic residues are shown as purple sticks, residues with regulatory function in green sticks and water molecules in red spheres. (B) A 2Fo-Fc composite omit electron density map contoured at 1.3 σ over the mean is shown surrounding the GSN peptide and Cys189 and Ser215. (C) Transpeptidation of the SFTI(N14)-GL peptide by the

S215A, E190K, and D147S variants of legumain, assayed at pH 6.0 and 0.05 μM enzyme concentration.

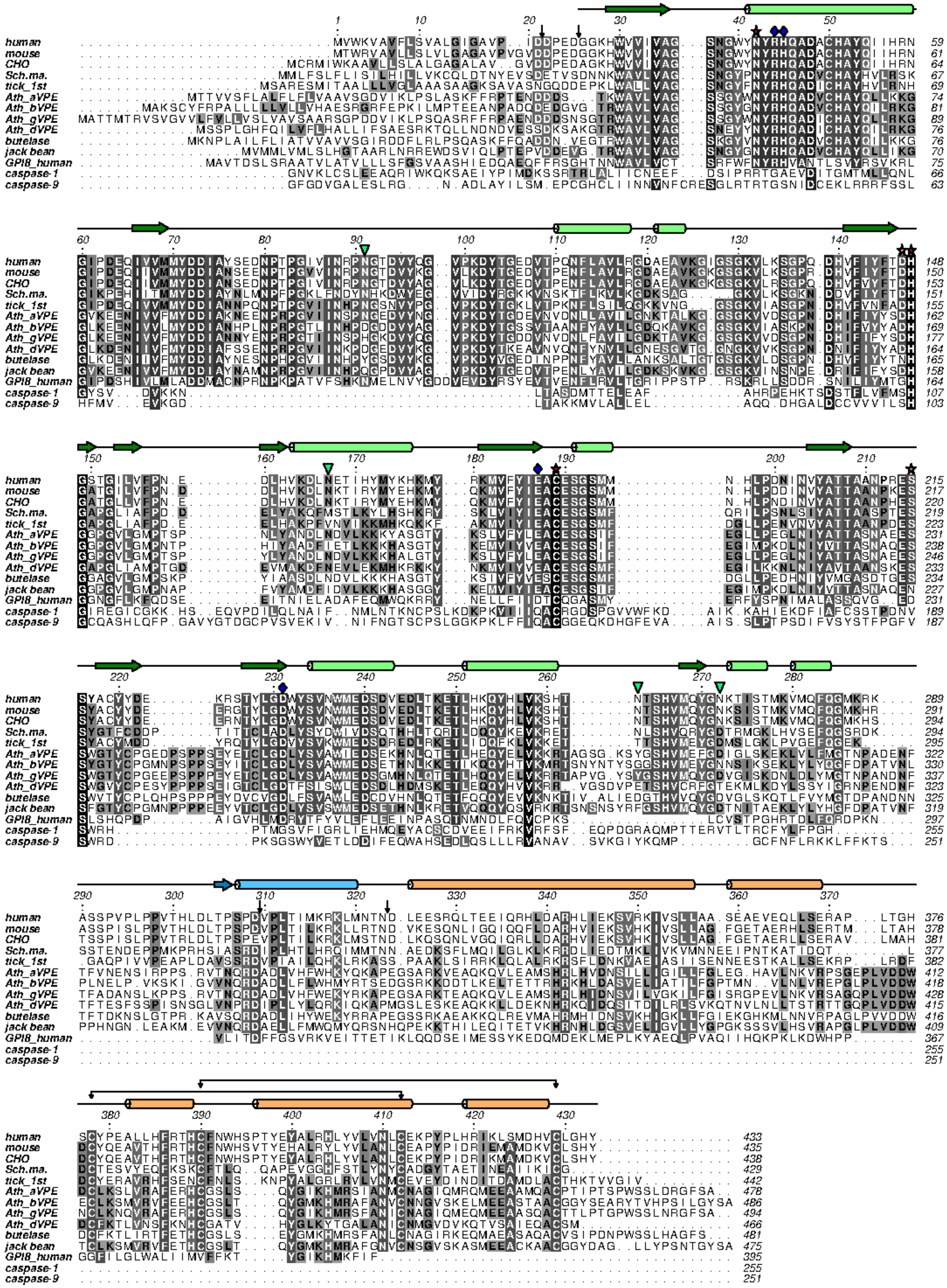
human
mouse
CHO
Sch.ma.
tick_1st
Ath_aVPE
Ath_bVPE
Ath_gVPE
Ath_dVPE
butelase
jack bean
GPI8_human
caspase-1
caspase-9

Figure S7. Sequence alignment of different clan CD proteases. Sequences of human (Uniprot ID Q99538), mouse (O89017), chinese hamster ovary (CHO, G3I1H5) and schistosome legumain (Q9NFY9), *Haemaphysalis longi*cornis legumain isoform 1 (tick_1st; A4PF00), *Arabidopsis thaliana* αVPE (P49047), βVPE (Q39044), γVPE (Q39119), δVPE (Q9LJX8), butelase 1 (*Clitoria ternatea*; A0A060D9Z7) and jack bean legumain (P49046), human GPI-transamidase (Q92643) and human caspase-1 (P29466) was prepared using ClustalW [17] and modified with Aline [18]. Autocatalytic processing sites are indicated with arrows, catalytic residues with red stars, residues close to the active site with pink stars, glycosylation sites with green triangles, S1-specificity residues with blue diamonds. The sequence numbering corresponds to human legumain. Secondary structure elements are indicated based on the crystal structure of human prolegumain (pdb entry 4fgu), whereby green elements correspond to the catalytic domain, blue elements to the activation peptide and orange elements to the LSAM (Legumain Stabilization and Activity Modulation) domain.

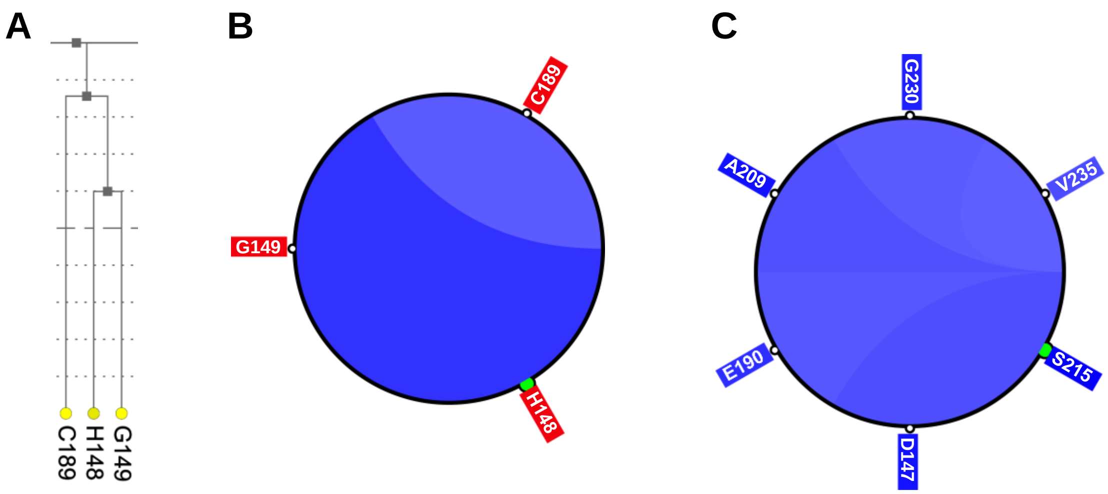

Figure S8. Covariance analysis reveals specific clusters of catalytic and regulatory amino acids. (A) A fragment of the cluster tree reveals that the catalytic Cys189 and His148 residues form, together with Gly149, which is the major constituent of the oxyanion hole, a catalytic site cluster. (B) Analysis of the closest relationships for His148 after applying a ≥ 0.3 cutoff to χ^2 scores further revealed Cys189 and Gly149 as the closest relatives. (C) Ser215, Asp147 and Glu190 form a cluster of regulatory residues.

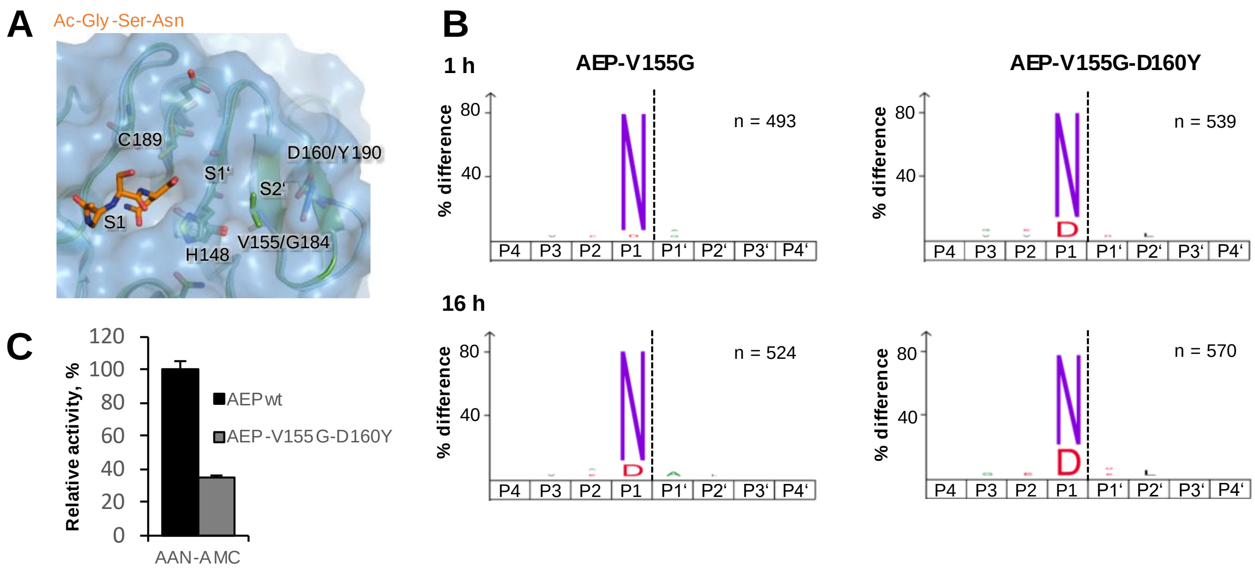

Figure S9. Grafting a plant-legumain-like S2' pocket on human legumain introduces specificity for Leu in P2' position. (A) Superposition of human legumain (green cartoon) in complex with the Gly-Ser-Asn peptide (orange sticks) and *A. thaliana* legumain isoform γ (AtAEPγ, blue cartoon). AtAEPγ is additionally shown as blue, transparent surface. Catalytic residues Cys189 and His148 are shown as sticks. Residues forming the S2' specificity pocket are V155 and D160 in human legumain and G184 and Y190 in AtAEPγ. (B) IceLogos of the V155G-legumain single mutant and the V155G-D160Y-legumain double mutant after 1 hour and 16 hours of incubation with the *E.coli* peptide library at pH 5.5 show a preference for Leu in P2' for the double mutant (p = 0.05). (C) Turnover of the AAN-AMC substrate by wild-type (AEPwt) and V155G-D160Y-legumain assayed at pH 5.5.

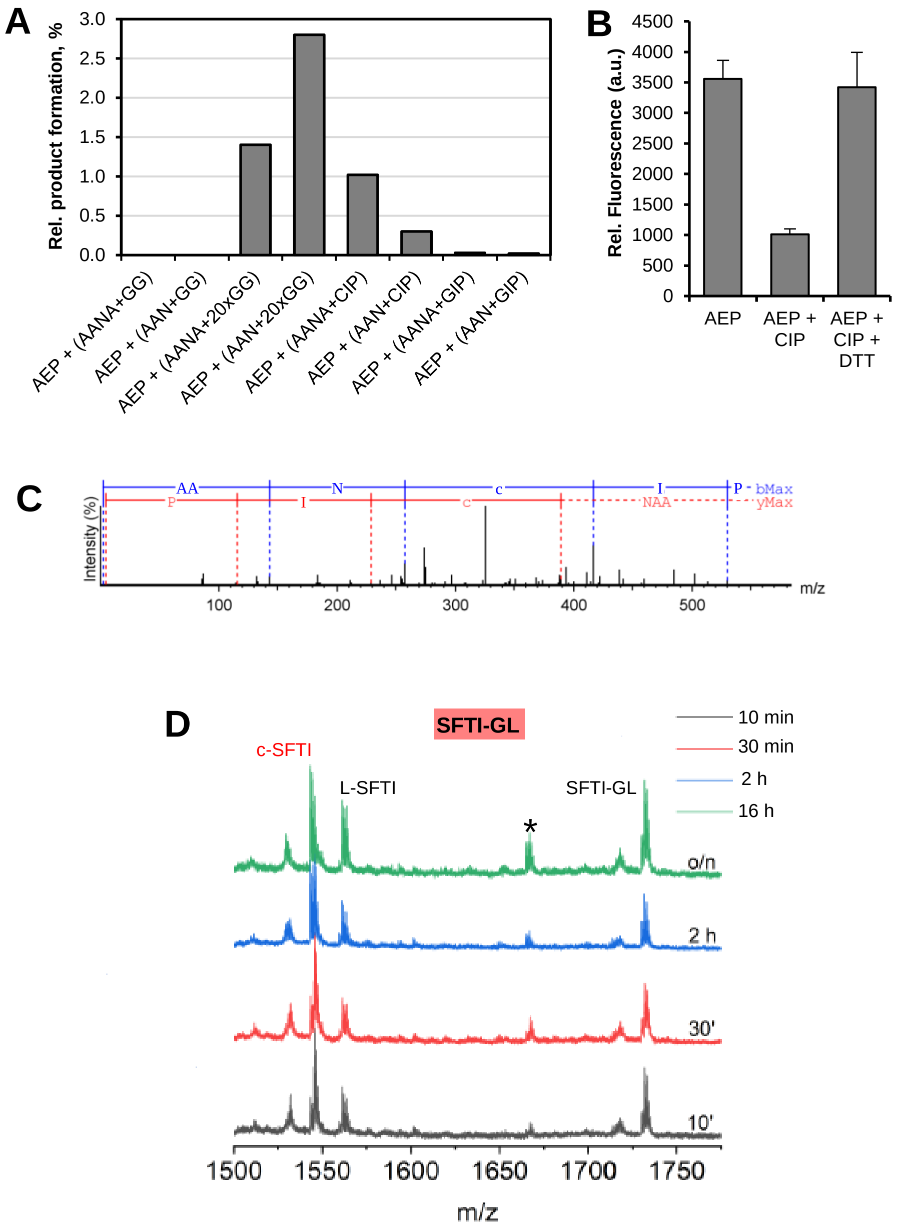

Figure S10. Legumain is ligase and transpeptidase towards linear peptides. (A) Relative formation of linked ligation product. Product formation was normalized to the peak area of the AAN, AANA, CIP or GIP peptides on the HPLC chromatogram before injection to the mass spectrometer. (B) Turnover of the AAN-AMC substrate at pH 5.5 after incubation of legumain with the CIP peptide in assay buffer with or without DTT as a reducing agent. (C) *De novo* sequencing of the collision induced fragment ions of the peptide AANCIP. The sample was treated with iodacetamide prior to mass spectrometry analysis. Addition of iodacetamide lead to alkylation

of cysteine residues and a subsequent increase in expected mass of 58 Da. A small letter “c” indicates alkylation (+58 Da) of the cysteine in the AANCIP peptide. (D) Time-series experiment showing conversion of the SFTI-GL precursor peptide to L-SFTI and c-SFTI after 10 min, 30 min, 2 h and 16 hours of incubation with legumain (10 µM) at pH 6.0. An asterisk is labelling an unknown species.

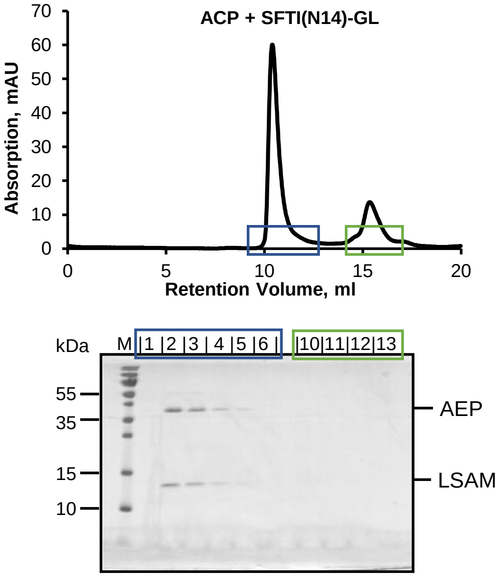

Figure S11. ACP remains a stable complex upon incubation with the SFTI(N14)-GL substrate. The two-chain state of legumain, which is a non-covalent complex formed by the AEP domain and the LSAM domain, was co-incubated with the SFTI(N14)-GL peptide (pH 6.0, 37 °C, 2 h) and the reaction was subsequently loaded on an S75 size exclusion column (pH 6.0). Fractions corresponding to the peaks were analyzed by SDS-PAGE and revealed co-migration of the AEP domain and the LSAM domain within the same peak.

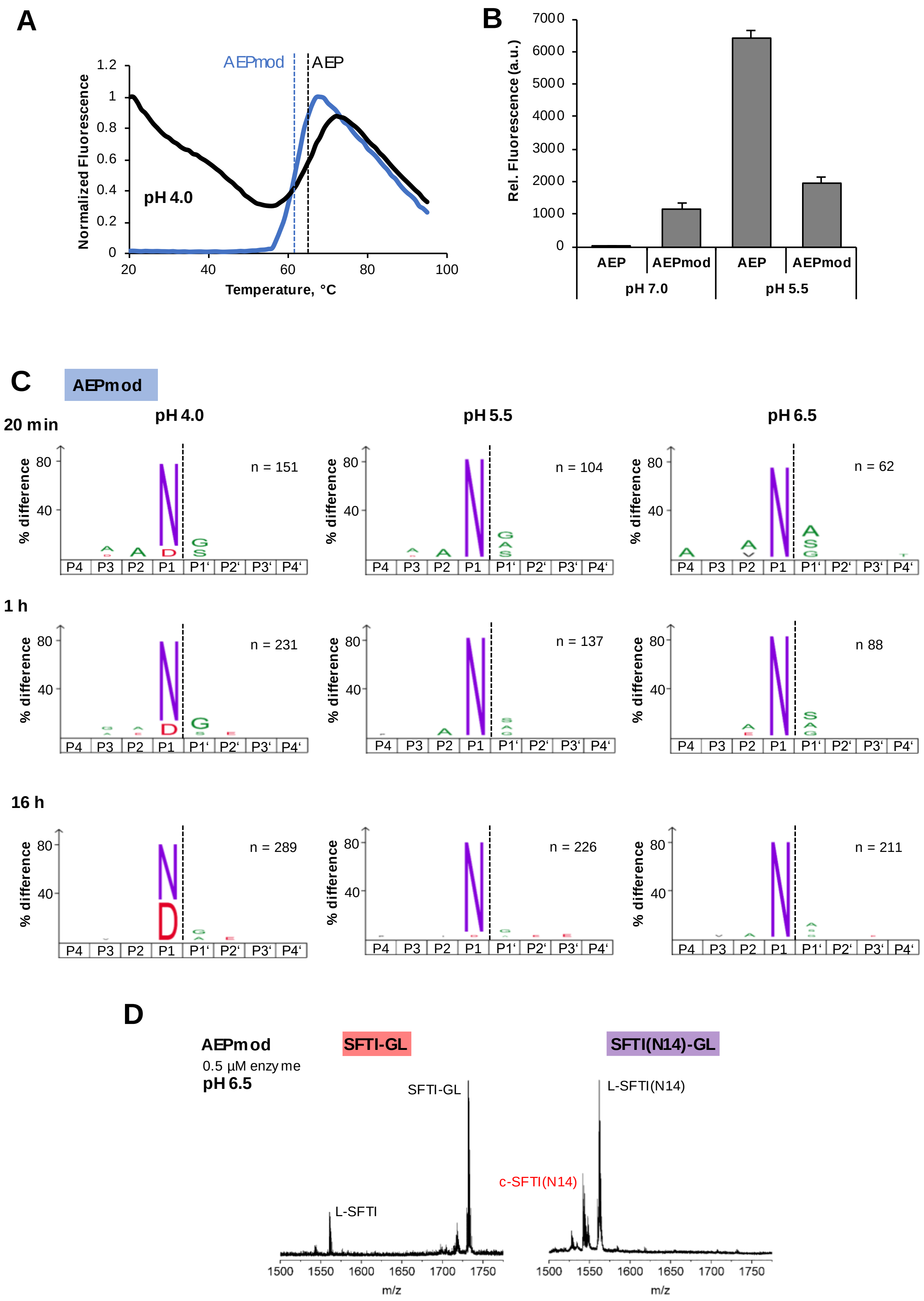
A
AEPmod
AEP
pH 4.0
Normalized Fluorescence
Temperature, °C
B
Rel. Fluorescence (a.u.)
AEP
AEPmod
AEP
AEPmod
pH 7.0
pH 5.5
C
AEPmod
20 min
pH 4.0
pH 5.5
pH 6.5
n = 151
n = 104
n = 62
1 h
n = 231
n = 137
n 88
16 h
n = 289
n = 226
n = 211
% difference
P4 P3 P2 P1 P1' P2' P3' P4'
D
AEPmod
0.5 µM enzyme
pH 6.5
SFTI-GL
SFTI(N14)-GL
SFTI-GL
L-SFTI
L-SFTI(N14)
c-SFTI(N14)
m/z

Figure S12. Chemical surface modification leads to stabilization of legumain. (A) The thermal stability of unmodified AEP and modified AEPmod was investigated using differential scanning fluorimetry and uncovered similar melting temperatures for both variants at pH 4.0. (B) The activity of legumain towards the AAN-AMC substrate was investigated at pH 7.0 and 5.5. While chemical modification led to a 40-fold increase in activity at pH 7.0, activity was reduced at pH 5.5. (C) IceLogos of AEPmod determined at pH 4.0, pH 5.5 and pH 6.5 after 20 min, 1 hour and 16 hours incubation of the enzyme with the peptide library respectively ($p = 0.05$). (D) Cyclisation of SFTI-GL and SFTI(N14)-GL peptides by AEPmod at pH 6.5 and 0.5 µM enzyme concentration.

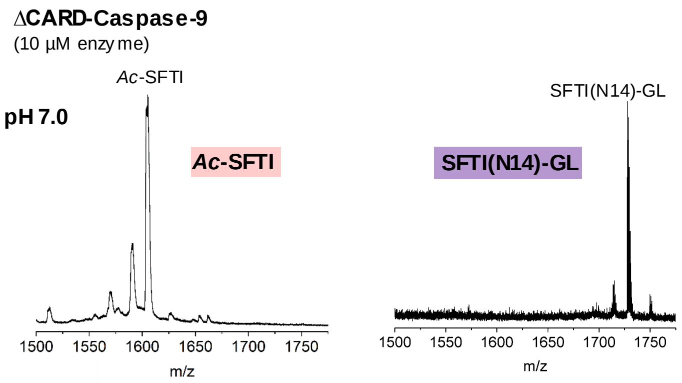

Figure S13. Caspase-9 is cyclising SFTI-derived precursor peptides. Cyclisation of the N-acetylated Ac-SFTI and the SFTI(N14)-GL control peptides was investigated at pH 7.0 and 10 µM enzyme concentration. Cyclisation was not observed for either substrates, confirming that the cyclization requires both the free N-terminus and Asp in P1 position.

Table S1. Cyclic product formed using indicated precursor peptides, enzyme concentrations and pH values (the % values are based on the relative abundance of the three species detected by mass spectrometry: precursor, processed linear and cyclic products). AtLEGγ-TC: *Arabidopsis thaliana* legumain isoform γ in two-chain activation state.

enzyme	pH	Conc. enzyme [µM]	SFTI-GL (%)	Conc enzyme [µM]	SFTI(N14)-GL (%)	Conc. enzyme [µM]	SFTI (%)	Conc. enzyme [µM]	SFTI(N14) (%)
**AEP**	4.0	0.5	**0**	0.5	**0**				
	6.0	0.5	**0**	0.5	**30**				
	6.0	10	**25**	0.05	**25**				
**AEP-S215A**	6.0	10	**10**	0.05	**10**				
**AEP-E190K**	6.0	10	**40**	0.05	**30**				
**AEP-D147S**	6.0	10	**0**	0.05	**10**				
**AEP-V155G-D160Y**	6.0	10	**60**	0.05	**30**				
**ACP**	6.0	10	**25**	0.5	**20**	10	**40**	10	**30**
**AEPmod**	6.0	0.5	**55**	0.5	**20**				
	6.0	10	**55**	0.05	**40**				
**Casp9**	6.0	10	**10**	10	**0**	10	**10**	10	**0**
**AEP**	6.5	10	**0**	0.05	**15**				
**AEPmod**	6.5	0.5	**0**	0.5	**30**				
**AtLEGγ**	6.0	0.5	**70**	0.5	**5**	0.5	**0**		
**AtLEGβ**	6.0	0.5	**60**	0.5	**40**	0.5	**15**	0.5	**40**
**AtLEGγ-TC**	6.0	0.5	**40**						

Table S2. Cyclisation-to-hydrolysis ratio (cyc:lin) determined using indicated precursor peptides, enzyme concentrations and pH values.

enzyme	pH	Conc. enzyme [μM]	SFTI-GL (cyc:lin)	Conc enzyme [μM]	SFTI(N14)-GL (cyc:lin)	Conc. enzyme [μM]	SFTI (cyc:lin)	Conc. enzyme [μM]	SFTI(N14) (cyc:lin)
**AEP**	4.0	0.5	**n.d.**	0.5	**n.d.**				
	6.0	0.5	**n.d.**	0.5	**0.7:1**	0.5	**n.d.**	0.5	**n.d.**
	6.0	10	**1.2:1**	0.05	**0.6:1**	10	**n.d.**	10	**n.d.**
**AEP-S215A**	6.0	10	**0.6:1**	0.05	**0.3:1**				
**AEP-E190K**	6.0	10	**0.7:1**	0.05	**0.6:1**				
**AEP-D147S**	6.0	10	**n.d.**	0.05	**0.2:1**				
**AEP-V155G-D160Y**	6.0	10	**1.3:1**	0.05	**0.9:1**				
**ACP**	6.0	10	**0.4:1**	0.5	**0.4:1**	10	**0.6:1**	10	**0.5:1**
**AEPmod**	6.0	0.5	**1.2:1**	0.5	**0.3:1**				
	6.0	10	**1.2:1**	0.05	**2:1**				
**Casp9**	6.0	10	**0.2:1**	10		10	**0.25:1**	10	
**AEP**	6.5	10	**n.d.**	0.05	**0.9:1**				
**AEPmod**	6.5	0.5	**n.d.**	0.5	**0.5:1**				

Table S3. Xray Data Collection and Refinement Statistics

	AEP-GSN
**Data collection**	
Space group	$P2_12_12_1$
Cell dimensions	
a, *b*, *c* (Å)	43.3, 75.1, 172.3
α, β, γ (°)	90, 90, 90
Resolution (Å)[a]	57.4–1.9 (1.94–1.90)
R_{merge}	0.11 (0.65)
R_{pim}	0.07 (0.47)
CC (1/2) (%)	0.99 (0.74)
$I/\sigma I$	9.6 (2.4)
Completeness (%)	99.5 (97.5)
Redundancy	4.2 (3.9)
**Refinement**	
Resolution (Å)	56.6–1.9
No. reflections	45086
R_{work} / R_{free}	19.2/21.9
No. atoms	
Protein	4247
Ligand/ion	137
Water	427
Overall B-factor (Å^2)	19.5
R.m.s deviations	
Bond lengths (Å)	0.01
Bond angles (°)	1.23

The structure was determined from a single crystal.

[a] Highest resolution shell is shown in parentheses.

References

1. Dall, E.; Brandstetter, H., Activation of legumain involves proteolytic and conformational events, resulting in a context- and substrate-dependent activity profile. *Acta Crystallogr F* **2012,** *68*, 24-31.
2. Dall, E.; Zauner, F. B.; Soh, W. T.; Demir, F.; Dahms, S. O.; Cabrele, C.; Huesgen, P. F.; Brandstetter, H., Structural and functional studies of Arabidopsis thaliana legumain beta reveal isoform specific mechanisms of activation and substrate recognition. *The Journal of biological chemistry* **2020,** *295* (37), 13047-13064.
3. Wang, J.; Wilkinson, M. F., Deletion mutagenesis of large (12-kb) plasmids by a one-step PCR protocol. *Biotechniques* **2001,** *31* (4), 722-4.
4. Denault, J.-B. S., Guy S., Expression, Purification, and Characterization of Caspases. *Current Protocols in Protein Science* **2002,** *21.13.1-21.13.15*.
5. Zauner, F. B.; Elsasser, B.; Dall, E.; Cabrele, C.; Brandstetter, H., Structural analyses of Arabidopsis thaliana legumain gamma reveal differential recognition and processing of proteolysis and ligation substrates. *The Journal of biological chemistry* **2018,** *293* (23), 8934-8946.
6. Niesen, F. *Excel script for the analysis of protein unfolding data acquired by Differential Scanning Fluorimetry (DSF)*, 3.0; Structural Genomics Consortium: Oxford, **2010**, 1-11.
7. Biniossek, M. L.; Niemer, M.; Maksimchuk, K.; Mayer, B.; Fuchs, J.; Huesgen, P. F.; McCafferty, D. G.; Turk, B.; Fritz, G.; Mayer, J.; Haecker, G.; Mach, L.; Schilling, O., Identification of Protease Specificity by Combining Proteome-Derived Peptide Libraries and Quantitative Proteomics. *Molecular & cellular proteomics : MCP* **2016,** *15* (7), 2515-24.
8. Schilling, O.; Huesgen, P. F.; Barre, O.; Auf dem Keller, U.; Overall, C. M., Characterization of the prime and non-prime active site specificities of proteases by proteome-derived peptide libraries and tandem mass spectrometry. *Nature protocols* **2011,** *6* (1), 111-20.
9. Dahms, S. O.; Demir, F.; Huesgen, P. F.; Thorn, K.; Brandstetter, H., Sirtilins - the new old members of the vitamin K-dependent coagulation factor family. *J Thromb Haemost* **2019,** *17* (3), 470-481.
10. Tyanova, S.; Temu, T.; Cox, J., The MaxQuant computational platform for mass spectrometry-based shotgun proteomics. *Nature protocols* **2016,** *11* (12), 2301-2319.
11. Colaert, N.; Helsens, K.; Martens, L.; Vandekerckhove, J.; Gevaert, K., Improved visualization of protein consensus sequences by iceLogo. *Nat Methods* **2009,** *6* (11), 786-7.
12. Battye, T. G.; Kontogiannis, L.; Johnson, O.; Powell, H. R.; Leslie, A. G., iMOSFLM: a new graphical interface for diffraction-image processing with MOSFLM. *Acta Crystallogr D Biol Crystallogr* **2011,** *67* (Pt 4), 271-81.
13. Winn, M. D.; Ballard, C. C.; Cowtan, K. D.; Dodson, E. J.; Emsley, P.; Evans, P. R.; Keegan, R. M.; Krissinel, E. B.; Leslie, A. G.; McCoy, A.; McNicholas, S. J.; Murshudov, G. N.; Pannu, N. S.; Potterton, E. A.; Powell, H. R.; Read, R. J.; Vagin, A.; Wilson, K. S., Overview of the CCP4 suite and current developments. *Acta Crystallogr D Biol Crystallogr* **2011,** *67* (Pt 4), 235-42.
14. McCoy, A. J.; Grosse-Kunstleve, R. W.; Adams, P. D.; Winn, M. D.; Storoni, L. C.; Read, R. J., Phaser crystallographic software. *J Appl Crystallogr* **2007,** *40* (Pt 4), 658-674.
15. Emsley, P.; Cowtan, K., Coot: model-building tools for molecular graphics. *Acta Crystallogr D Biol Crystallogr* **2004,** *60* (Pt 12 Pt 1), 2126-32.
16. Adams, P. D.; Grosse-Kunstleve, R. W.; Hung, L. W.; Ioerger, T. R.; McCoy, A. J.; Moriarty, N. W.; Read, R. J.; Sacchettini, J. C.; Sauter, N. K.; Terwilliger, T. C., PHENIX:

building new software for automated crystallographic structure determination. *Acta Crystallogr D Biol Crystallogr* **2002,** *58* (Pt 11), 1948-54.
17. Thompson, J. D.; Higgins, D. G.; Gibson, T. J., CLUSTAL W: improving the sensitivity of progressive multiple sequence alignment through sequence weighting, position-specific gap penalties and weight matrix choice. *Nucleic Acids Res* **1994,** *22* (22), 4673-80.
18. Bond, C. S.; Schuttelkopf, A. W., ALINE: a WYSIWYG protein-sequence alignment editor for publication-quality alignments. *Acta Crystallogr D Biol Crystallogr* **2009,** *65* (Pt 5), 510-2.
